# Bromomaleimide-Linked Bioconjugates Are Cleavable in Mammalian Cells

**DOI:** 10.1002/cbic.201100603

**Published:** 2011-11-18

**Authors:** Paul Moody, Mark E B Smith, Chris P Ryan, Vijay Chudasama, James R Baker, Justin Molloy, Stephen Caddick

**Affiliations:** [a] Department of Chemistry, University College LondonGordon Street, London, WC1H 0AJ (UK); [b] MRC National Institute of Medical Research, The RidgewayMill Hill, London, NW7 1AA (UK)

**Keywords:** drug delivery, fluorescent probes, in-cell cleavage, microinjection, protein modifications

Bromomaleimides have recently been shown to act as small, versatile scaffolds for the controlled assembly of thiolated biomolecules.[1–4] They present three points of chemical attachment, thereby allowing the modular construction of multifunctional bioconjugates. The bromomaleimide scaffold introduces no new chiral centres to the final construct and, in contrast to disulfide linkages, bromomaleimides can be used to link two unactivated biomolecules of equal concentration with minimal formation of homodimeric side products.^2^ Bromomaleimide adducts have been shown to dissociate in vitro in the presence of reducing agents to liberate the composite thiols.^2^ The cytoplasm of cells contains 1–10 mm reduced glutathione,^5^ thus raising the possibility that bromomaleimide conjugates could be cleavable in vivo. If this could be demonstrated, then a number of medical and academic applications can be envisaged that combine in vivo cleavage with multiple points of scaffold attachment. However, a number of factors might inhibit cytoplasmic cleavage, in particular pH-dependant^3^ and protease-catalysed amide bond hydrolysis. Using a series of bromomaleimide-linked green fluorescent protein (GFP)–rhodamine conjugates, designed as FRET pairs, we demonstrate herein that bromomaleimide-linked bioconjugates cleave in the cytoplasm of mammalian cells.

Rhodamine–maleimide derivatives **4**–**6** were generated by condensation of the relevant maleic anhydride with a common intermediate, **3** (Scheme 1).

**Scheme 1:**
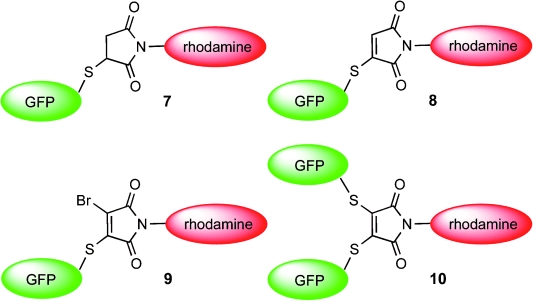
Synthesis of rhodamine-bromomaleimides. a) (COCl)_2_, 20 °C, 15 h; b) piperidin-4-yl carbamic acid *tert*-butyl ester (10.4 equiv), CsCO_3_ (10.4 equiv), CH_2_Cl_2_, 20 °C, 24 h, 71 % (2 steps); c) TFA/CH_2_Cl_2_ (1:1), 20 °C, 5 h, 100 %; d) maleic anhydride (1.4 equiv), AcOH, 120 °C, 5 h, 40 %; e) bromomaleic anhydride (1.4 equiv), AcOH, 120 °C, 5 h, 66 %; f) dibromomaleic anhydride (1.4 equiv), AcOH, 120 °C, 5 h, 66 %.

The two native cysteines in wild-type superfolder GFP,^6^ C48 and C70, were shown to be inaccessible to maleimide functionalisation under our reaction conditions (see Section 4 in the Supporting Information). A GFP with a free, accessible thiol close to its fluorophore (GFP-SH) was generated by introducing an S147C mutation into superfolder GFP. The mutated GFP-SH produces a similar emission spectrum to wild-type superfolder GFP. The folded protein was shown to be resistant to disulfide-mediated dimerisation, thus allowing GFP-SH to be conjugated to maleimides without the need for reducing agents (see Section 5 in the Supporting Information).

Compounds **4**–**6** were attached to GFP-SH as described in the Supporting Information, Section 5. Stoichiometric addition of rhodamine–maleimide was confirmed by mass spectrometry. The emission spectra of the resultant constructs **7**–**10** are illustrated in Figure 1. In each case, addition of rhodamine–maleimide was shown to result in efficient quenching of GFP fluorescence. Little increase in emission at 590 nm was seen.


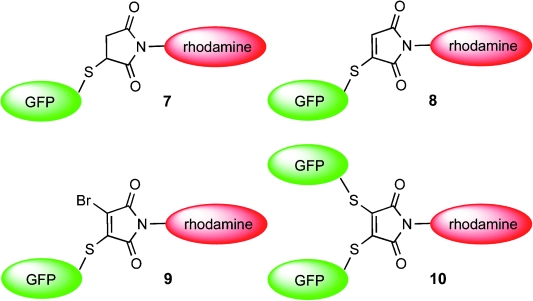


**Figure 1:**
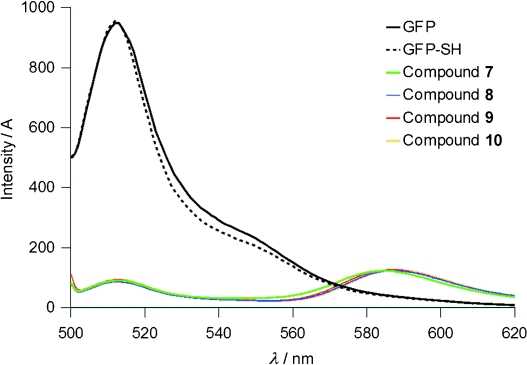
Emission spectra of superfolder GFP, the mutant GFP-SH and the rhodamine conjugates; compounds **7**–**9** (0.85 μM), compound **10** (0.425 μM; *λ*_ex_=494 nm).

Cleavage of compounds **7**–**10** by a physiologically relevant concentration of reduced glutathione (1 mM) was monitored in vitro by dual-channel measurement of the GFP and rhodamine emission intensities upon excitation of GFP at 494 nm (Figure 2).

**Figure 2:**
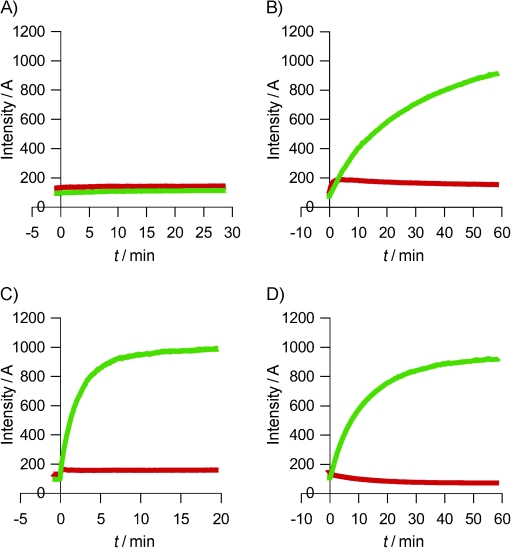
In vitro cleavage of the rhodamine–GFP conjugates. Glutathione (1 mM) was added to compounds A) **7**, B) **8**, C) **9** (0.85 μM each) and D) **10** (0.43 μM). GFP was excited at 494 nm; GFP emission (515 nm, green) and rhodamine emission (590 nm, red) were measured simultaneously.

As expected, the GFP emission intensity associated with **7** did not change upon addition of glutathione, whereas that associated with **8**–**10** increased. The data fitted well to a single exponential increase in free GFP concentration over time, suggesting first-order kinetics with respect to conjugates **8**–**10** under these conditions. Rate constants of 1.9 s^−1^ for **8**, 26 s^−1^ for **9** and 2.8 s^−1^ for **10** were calculated.

For compounds **7**–**9**, little change in apparent rhodamine emission (590 nm) was seen during the experiments. For compound **10**, a decrease in rhodamine emission was observed; this suggesting that FRET is disrupted. The efficient quenching of GFP fluorescence allowed us to use the ratio of GFP/rhodamine emission intensities as a quantitative measure of cleavage during subsequent microinjection experiments. Cleavage of constructs **8**–**10** was also confirmed by mass spectrometry under analogous reaction conditions (see Section 7 in the Supporting Information).

Cleavage of constructs **8**–**10** in human cells was demonstrated by microinjection into live, cultured HeLa cells, followed by time-lapse fluorescence microscopy. GFP was excited by using a 450–490 nm filter, and GFP and rhodamine emission were measured simultaneously by using an image splitter that contained GFP (500–550 nm) and rhodamine (575–630 nm) emission filters (Figures 3 and 4).

**Figure 3:**
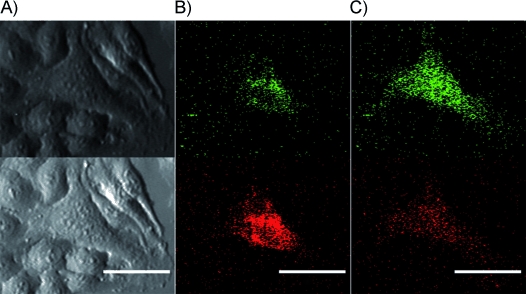
In-cell cleavage of compound **9** following microinjection into HeLa cells. The image was cast onto the upper and lower halves of the CCD sensor by using an image splitter (Optical Insights). Top: GFP emission, bottom: rhodamine emission. A) DIC image immediately before injection. B) Fluorescence image immediately after injection. C) Fluorescence image 10 min after cleavage. Scale bars=5.0 μm.

**Figure 4:**
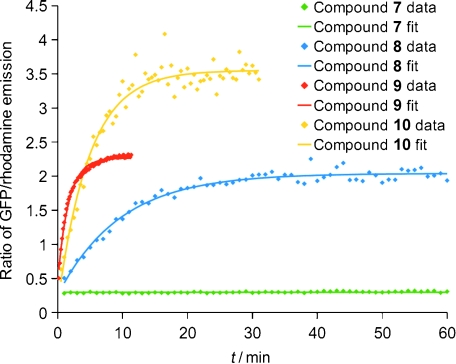
In-cell cleavage of compounds **7**–**10** in HeLa cells. The GFP/rhodamine emission ratio and exponential fits were calculated as described in the Supporting Information.

In each case, both the absolute GFP intensity post-microinjection (see Section 8 in the Supporting Information) and ratiometric data fitted well to a single exponential increase, thus showing that bromomaleimide-linked constructs are cleaved in cells. The order of reactivity is the same as that observed in vitro (see Section 9 in the Supporting Information); this suggests that the mechanism of cleavage in cells is the same as the mechanism in vitro. The ratio of GFP/rhodamine emission intensity at equilibrium is consistent with the ratio observed for cleaved constructs deposited directly onto glass slides (see Section 10 in the Supporting Information). This suggests that complete cleavage of the bromomaleimide linkages occurs in HeLa cells. Disassembly of the fully conjugated dibromomaleimide (**10**) was also successfully demonstrated in COS-7 cells (see Section 8 in the Supporting Information), thus highlighting that observed in cell cleavage is not restricted to HeLa cells.

In summary, we have demonstrated that bromomaleimide-linked conjugates can be cleaved with first-order kinetics both in vitro and in cells. Dibromomaleimide derivatives (**9** and **10**) cleave at a faster rate than monobromomaleimide construct **8**. Maleimide construct **7** is stable to thiol-promoted cleavage. We envisage that bromomaleimides have potential as scaffolds for the synthesis of a variety of multifunctional reagents. We believe that the FRET construct **9** has a specific application in the screening of attached thiolated cell-penetrating structures capable of successfully delivering protein cargo to the cytoplasm. More broadly, we believe that bromomaleimide scaffolds provide a potential core structure for the facile and versatile construction of targeted therapeutics that will cleave to release their cargo once internalised into the cytoplasm. We shall report on the further development and application of this methodology in due course.

## Supporting Information

Detailed facts of importance to specialist readers are published as ”Supporting Information”. Such documents are peer-reviewed, but not copy-edited or typeset. They are made available as submitted by the authors.
