# Supplementary material for: Bromomaleimide-Linked Bioconjugates Are Cleavable in Mammalian Cells
Source: Chembiochem. 2011 Nov 18;13(1):39–41. doi: 10.1002/cbic.201100603 (PMC3320661; doi:10.1002/cbic.201100603)
Supplement: Supplementary file 1 [file cbic0013-0039-SD1.pdf]

CHEMBIOCHEM

Supporting Information

© Copyright Wiley-VCH Verlag GmbH & Co. KGaA, 69451 Weinheim, 2011

Bromomaleimide-Linked Bioconjugates Are Cleavable in Mammalian Cells

Paul Moody,[a, b] Mark. E. B. Smith,[a] Chris P. Ryan,[a] Vijay Chudasama,[a] James R. Baker,*[a] Justin Molloy,*[b] and Stephen Caddick*[a]

cbic_201100603_sm_miscellaneous_information.pdf

SUPPORTING INFORMATION

Bromomaleimide-linked bioconjugates are cleavable in mammalian cells

Paul Moody,[a,b] Mark E. B. Smith,[a] Chris P. Ryan,[a] Vijay Chudusama,[a] James R. Baker,[a] Justin Molloy,[b] Stephen Caddick*[a]

a. Department of Chemistry, UCL, Gordon Street, London, WC1H 0AJ, U.K.
b. MRC National Institute of Medical Research, The Ridgeway, Mill Hill, London NW7 1AA, U.K.

1. General Procedures

^{1}H and ^{13}C NMR spectra were recorded at room temperature on a Bruker Avance 600 instrument operating at a frequency of 600 MHz for ^{1}H and 150 MHz for ^{13}C. ^{1}H NMR spectra were referenced to the $CDCl_3$ (7.26 ppm) signal. ^{13}C NMR spectra were referenced to the $CDCl_3$ (77.67 ppm) signal. Mass spectra and high resolution mass data for small molecules were recorded on a VG70-SE mass spectrometer (EI mode). Rhodamine-B, maleic anhydride and bromomaleic ahnydride were purchased from Sigma-Aldrich and used without further purification. Dibromomaleic anhydride was synthesized as described previously.[1]

2. Protein Mass Spectrometry

LC-MS was performed on protein samples using a Waters Acquity uPLC connected to Waters Acquity Single Quad Detector (SQD). Column: Acquity uPLC BEH C18 1.7µm 2.1 x 50 mm. Wavelength: 254 nm. Mobile Phase: 95:5 Water (0.1% Formic Acid): MeCN (0.1% Formic Acid) Gradient over 4 min (to 5:95 Water (0.1% Formic Acid): MeCN (0.1% Formic Acid). Flow Rate: 0.6 mL/min. MS Mode: ES+. Scan Range: m/z = 85-2000. Scan time: 0.25 sec. Data obtained in continuum mode. The electrospray source of the MS was operated with a capillary voltage of 3.5 kV and a cone voltage of 50 V. Nitrogen was used as the nebulizer and desolvation gas at a total flow of 600 L/h.Total mass spectra for protein samples were reconstructed from the ion series using the MaxEnt 1 algorithm pre-installed on MassLynx software.

3. Synthesis of Rhodamine-Maleimide Reagents.

(9-(2-(4-*tert*-Butoxycarbonylamino-piperidine-1-carbonyl)-phenyl)-6-diethylamino-xanthen-3-ylidene)-diethyl-ammonium chloride 2

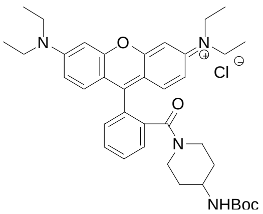

Rhodamine B (50 mg, 0.10 mmol) was dissolved in oxalyl chloride (10 mL) and the reaction mixture stirred under argon for 15 h. Excess thionyl chloride was removed *in vacuo* and the residue dissolved in CH_2Cl_2 (5 mL). The rhodamine acid chloride in CH_2Cl_2 was then added dropwise to a stirred solution of piperidin-4-yl-carbamic acid *tert*-butyl ester (209 mg, 1.04 mmol) and Cs_2CO_3 (340 mg, 104 mmol) in CH_2Cl_2 (5 mL) and the reaction mixture stirred for a further 24 h, after which the CH_2Cl_2 was removed *in vacuo* to give a purple solid. The crude product was purified by column chromatography (gradient 2-10% MeOH/CH_2Cl_2) to yield the desired product as a purple solid (46 mg, 0.07 mmol, 71% yield). ^{1}H NMR (600 MHz, CD_3OD) δ 7.76 (m, 2H), 7.66 (dd, J = 2.5, 7.0 Hz, 1H), 7.50 (dd, J = 2.5, 7.0 Hz, 1H), 7.28 (dd, J = 5.0, 9.0 Hz, 2H), 7.08 (t, J = 9.0 Hz, 2H), 6.98 (d, J = 2.5 Hz, 2H), 4.08 (d, J = 13.0 Hz, 1H), 3.73 (bs, 1H), 3.70 (q, J = 7.0 Hz, 8H), 3.46 (m, 1H), 3.05 (bs, 1H), 2.68 (bs, 1H), 1.76 (t, J = 14.5 Hz, 2H), 1.41 (s, 9H), 1.32 (t, J = 7.0 Hz, 12H), 1.18 (bs, 2H). ^{13}C NMR (150 MHz, CD_3OD) δ 169.3 (s), 159.3 (s), 157.6 (s), 157.2 (s), 157.1 (s), 137.1 (s), 133.3 (d), 132.0 (d), 131.7 (d), 131.3 (d), 131.1 (d), 128.6 (d), 115.5 (d), 115.3 (d), 114.9 (s), 114.8 (s), 97.4 (d), 80.1 (s), 41.8 (t), 33.5 (t), 32.3 (t), 28.8 (q), 12.9 (q). LRMS (TOF MS ES+) $[M]^+$ 625.3748, 100%. HRMS (TOF MS ES+) calcd for $C_{38}H_{49}N_4O_4$ $[M]^+$ 625.3754, observed 625.3748.

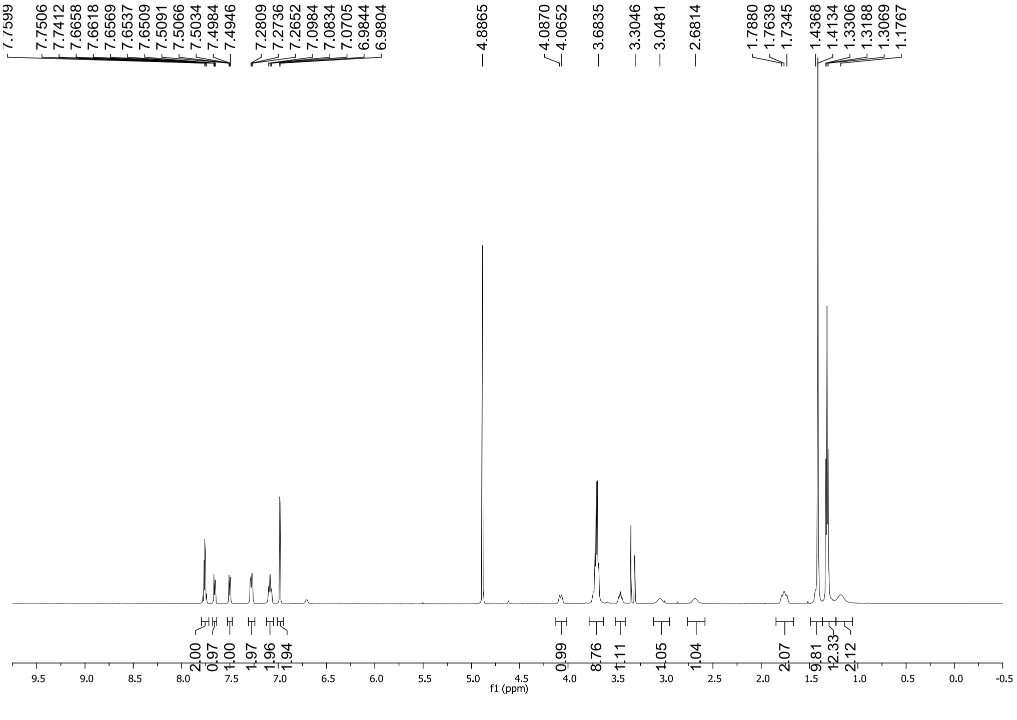

{9-[2-(4-Amino-piperidine-1-carbonyl)-phenyl]-6-diethylamino-xanthen-3-ylidene}-diethyl-ammonium chloride, trifluoroacetate salt 3

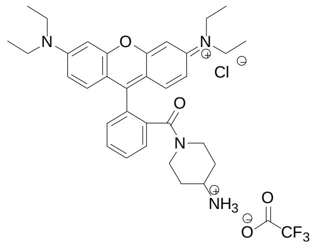

TFA (5 mL) was added to a stirred solution of carbamate **2** (46 mg, 0.07 mmol) in CH_2Cl_2 (5 mL) and the reaction mixture stirred for 5 h. The solvent was azeotropically removed using toluene (2 x 10 mL) to yield the desired product as a purple solid (37 mg, 0.07 mmol, 100%) which was used without further purification. ^{1}H NMR (600 MHz, CD_3OD) δ 7.77 (m, 2H), 7.66 (dd, J = 2.0, 7.5 Hz, 1H), 7.50 (dd, J = 2.0, 7.5 Hz, 1H), 7.27 (d, J = 9.5 Hz, 2H), 7.07 (t, J = 10.5 Hz, 2H), 6.82 (d, J = 2.0 Hz, 2H), 4.30 (d, J = 12.5 Hz, 1H), 3.84 (d, J = 12.5 Hz, 1H), 3.68 (m, 8H), 3.32 (m, 1H), 2.98 (bs, 1H), 2.57 (bs, 1H), 1.96 (d, J = 12.0 Hz, 2H), 1.41 (bs, 2H), 1.33 (t, J = 7.0 Hz, 12H). ^{13}C NMR (150 MHz, CD_3OD) δ 169.6 (s), 159.3 (s), 157.3 (s), 157.2 (s), 156.7 (s), 136.7 (s), 133.4 (d), 133.0 (d), 131.9 (d), 131.8 (s), 131.4 (d), 131.3 (d), 129.2 (d), 128.4 (d), 127.8 (d), 115.4 (d), 115.3 (d), 114.6 (s), 97.4 (d), 41.1 (t), 31.3 (t), 30.5 (t), 12.8 (q). LRMS (TOF MS ES+) $[M]^+$ 525.3234, 100%. HRMS (TOF MS ES+) calcd for $C_{33}H_{44}N_4O_2$ $[M]^+$ 525.3230, observed 525.3234.

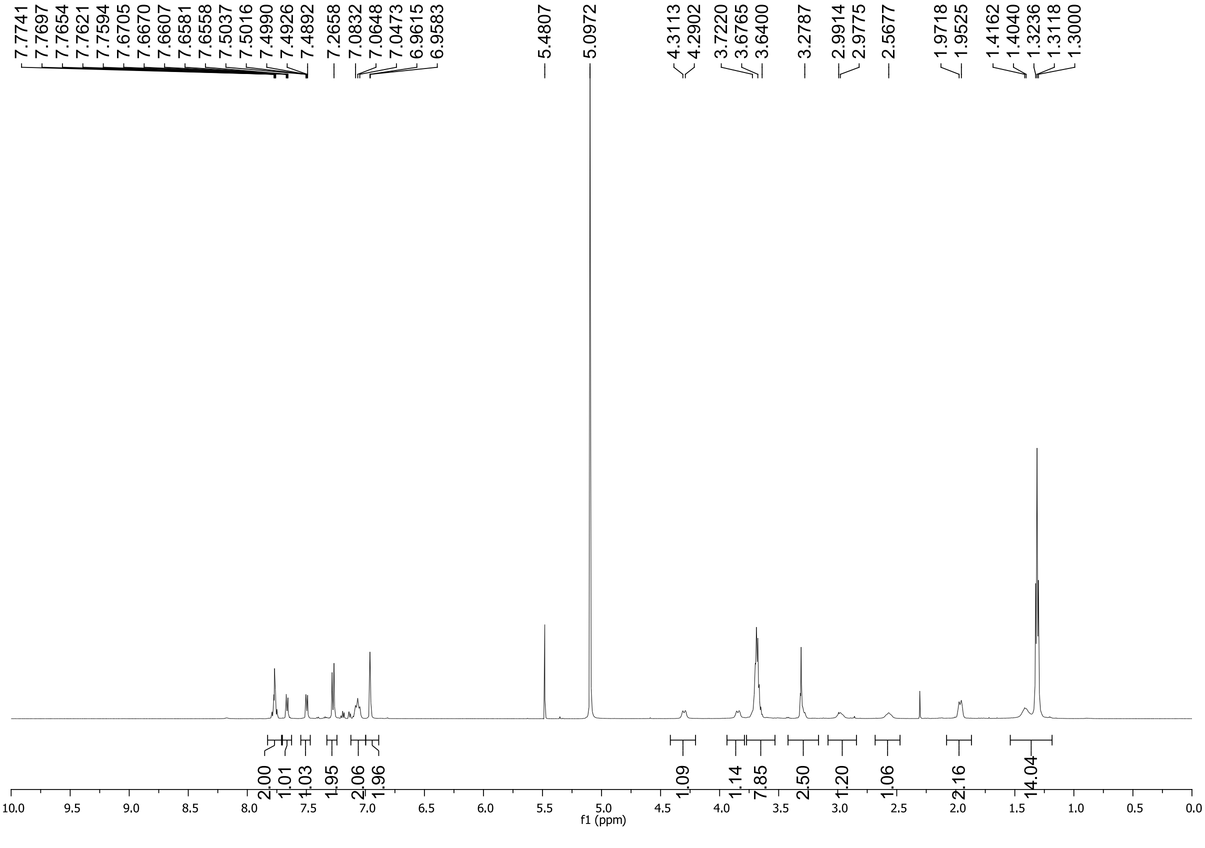

General synthesis for rhodamine maleimide derivatives

Maleic anhydride, bromomaleic anhydride or dibromomaleic anhydride (1.4 eq) was added in one portion to rhodamine trifluoroacetate salt **3** (1 eq) in AcOH (0.05 mL mg^{-1}) and the reaction mixture heated to 120 °C for 5 h. Following this, the AcOH was azeotropically removed using toluene to give crude product, which was purified by column chromatography (gradient 2-20% MeOH/CH_2Cl_2) to yield product as a purple solid.

(6-Diethylamino-9-(2-(4-(2,5-dioxo-2,5-dihydro-pyrrol-1-yl)-piperidine-1-carbonyl)-phenyl)-xanthen-3-ylidene)-diethyl-ammonium chloride 4.

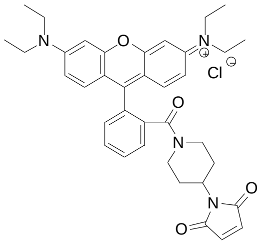

40% yield. ^{1}H NMR (600 MHz, CD_3OD) δ 7.76 (m, 2H), 7.65 (m, 1H), 7.51 (m, 1H), 7.28 (m, 2H), 7.06 (dd, J = 2.0, 9.5 Hz, 2H), 6.99 (m, 2H), 6.74 (s, 2H), 4.36 (d, J =13.0 Hz, 1H), 4.04 (m, 1H), 3.84 (d, J = 13.0 Hz, 1H), 3.69 (m, 8H), 3.11 (bs, 1H), 2.61 (m, 1H), 1.78 (bs, 2H), 1.55 (m, 1H), 1.45 (m, 1H), 1.31 (m, 12H). ^{13}C NMR (150 MHz, CD_3OD) δ 175.4 (s), 175.1 (s), 171.9 (s), 171.6 (s), 169.3 (s), 157.4 (s), 156.8 (s), 137.0 (s), 132.6 (d), 132.0 (d), 131.3 (d), 131.1 (d), 128.7 (d), 117.3 (d), 116.0 (d), 115.1 (d), 114.8 (s), 97.7 (d), 97.2 (d), 69.2, 42.5 (t), 36.0 (t), 28.4 (t), 20.5 (t), 12.9 (q). LRMS (TOF MS ES+) $[M]^+$ 605.312, 100%. HRMS (TOF MS ES+) calcd for $C_{37}H_{41}N_4O_4$ $[M]^+$ 605.3128, observed 605.3120.

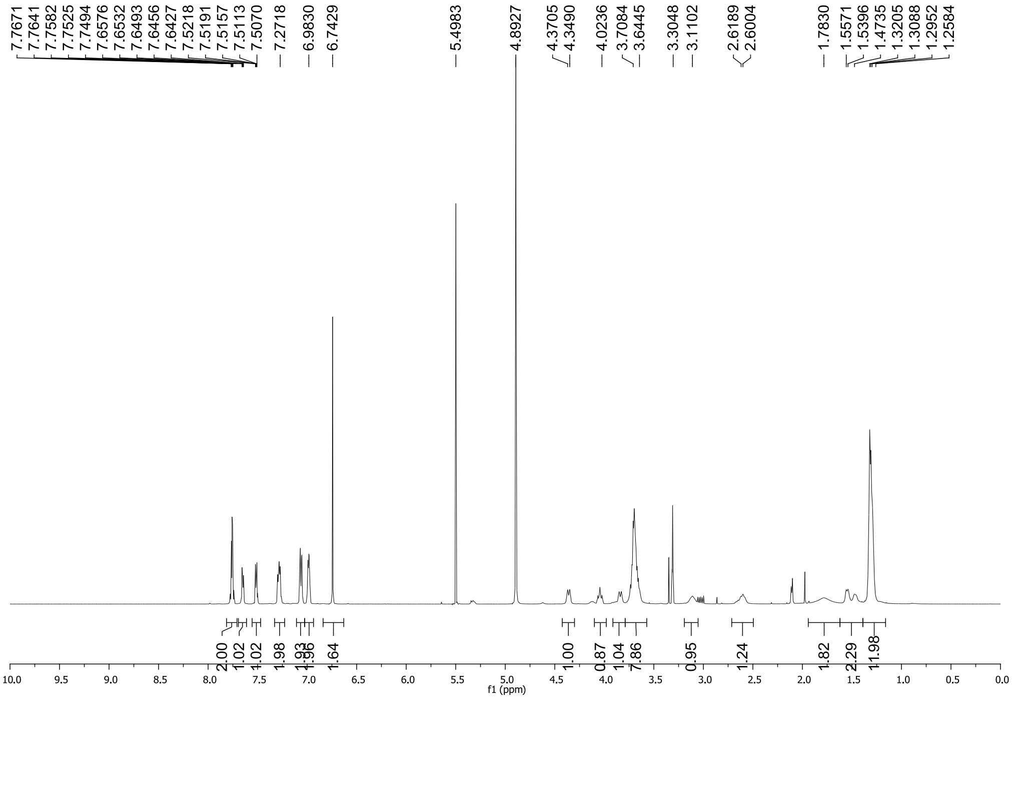

(9-(2-(4-(3-Bromo-2,5-dioxo-2,5-dihydro-pyrrol-1-yl)-piperidine-1-carbonyl)-phenyl)-6-diethylamino-xanthen-3-ylidene)-diethyl-ammonium chloride 5.

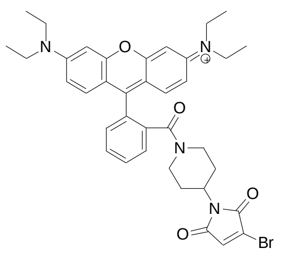

66% yield. ^{1}H NMR (600 MHz, CD_3OD) δ 7.78 (m, 2H), 7.66 (m, 1H), 7.53 (m, 1H), 7.30 (m, 2H), 7.09 (m, 3H), 7.00 (s, 2H), 4.38 (d, J = 12.5 Hz, 1H), 4.10 (m, 1H), 3.88 (d, J = 13.0 Hz, 1H), 3.69 (m, 8H), 3.17 (t, J = 12.0 Hz, 1H), 2.64 (t, J = 12.0 Hz, 1H), 1.75 (d, J = 8.0 Hz, 2H), 1.58 (m, 2H), 1.33 (m, 12H). ^{13}C NMR (150 MHz, CD_3OD) δ 168.1 (s), 167.9 (s), 157.9 (s), 155.9 (s), 155.5 (s), 135.6 (d), 132.0(s), 131.0 (d), 130.8 (d), 130.7 (d), 130.2 (d), 129.8 (d), 129.7 (d), 127.4 (d), 113.8 (s), 96.3 (d), 95.8 (d), 45.5 (t), 45.4 (t), 41.1 (t), 29.0 (t), 11.5 (q). LRMS (TOF MS ES+) $[M]^+$ 683.233, 100%. HRMS (TOF MS ES+) calcd for $C_{37}H_{40}N_4O_4Br$ $[M]^+$ 683.2233, observed 683.2233.

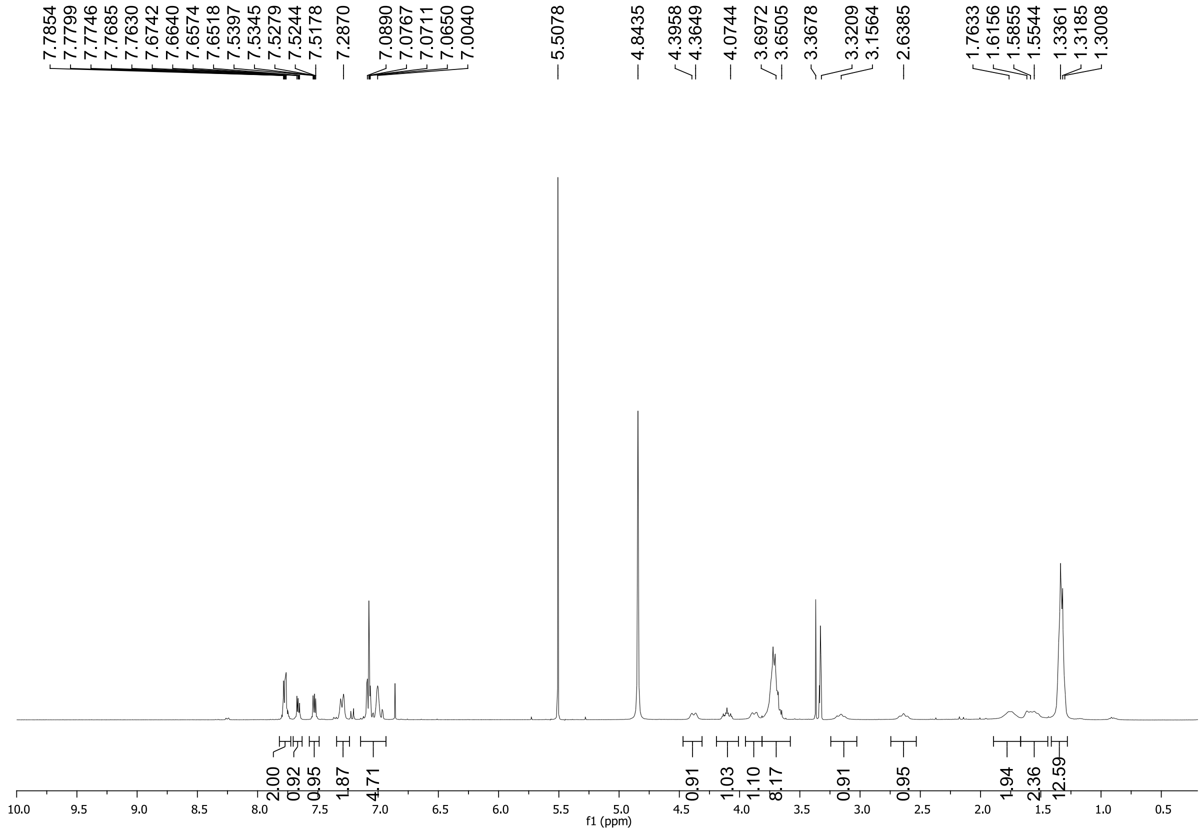

(9-(2-(4-(3,4-Dibromo-2,5-dioxo-2,5-dihydro-pyrrol-1-yl)-piperidine-1-carbonyl)-phenyl)-6-diethylamino-xanthen-3-ylidene)-diethyl-ammonium chloride 6.

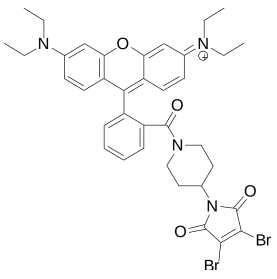

62% yield. ^{1}H NMR (600 MHz, CD_3OD) δ 7.76 (m, 2H), 7.65 (m, 1H), 7.51 (m, 1H), 7.28 (m, 2H), 7.06 (d, J = 9.5 Hz, 2H), 7.00 (d, J = 12.5 Hz, 2H), 4.63 (bs, 1H), 4.37 (d, J = 13.0 Hz, 1H), 4.14 (m, 1H), 3.89 (d, J = 13.0 Hz, 1H), 3.73 (m, 8H), 3.18 (bs, 1H), 2.65 (bs, 1H), 1.63 (bs, 1H), 1.57 (bs, 2H), 1.32 (t, J = 7.0 Hz, 12H). ^{13}C NMR (150 MHz, CD_3OD) δ 169.3 (s), 164.8 (s), 164.3 (s), 163.8 (s), 159.3 (s), 159.3 (s), 157.4 (s), 157.2 (s), 156.9 (s), 138.7 (s), 136.9 (s), 134.0 (d), 132.3 (d), 131.6 (d), 131.2 (d), 131.1 (d), 128.8 (d), 116.2 (d), 115.0 (d), 114.7 (s), 97.7 (d), 51.0 (d), 50.9 (d), 47.0 (t), 42.4 (t), 30.4 (t), 29.6 (t), 12.9 (q). LRMS (TOF MS ES+) $[M]^+$, 761.1264, 100%. HRMS (TOF MS ES+) calcd for $C_{37}H_{39}N_4O_4Br_2$ $[M]^+$ 761.1338, observed 761.1354.

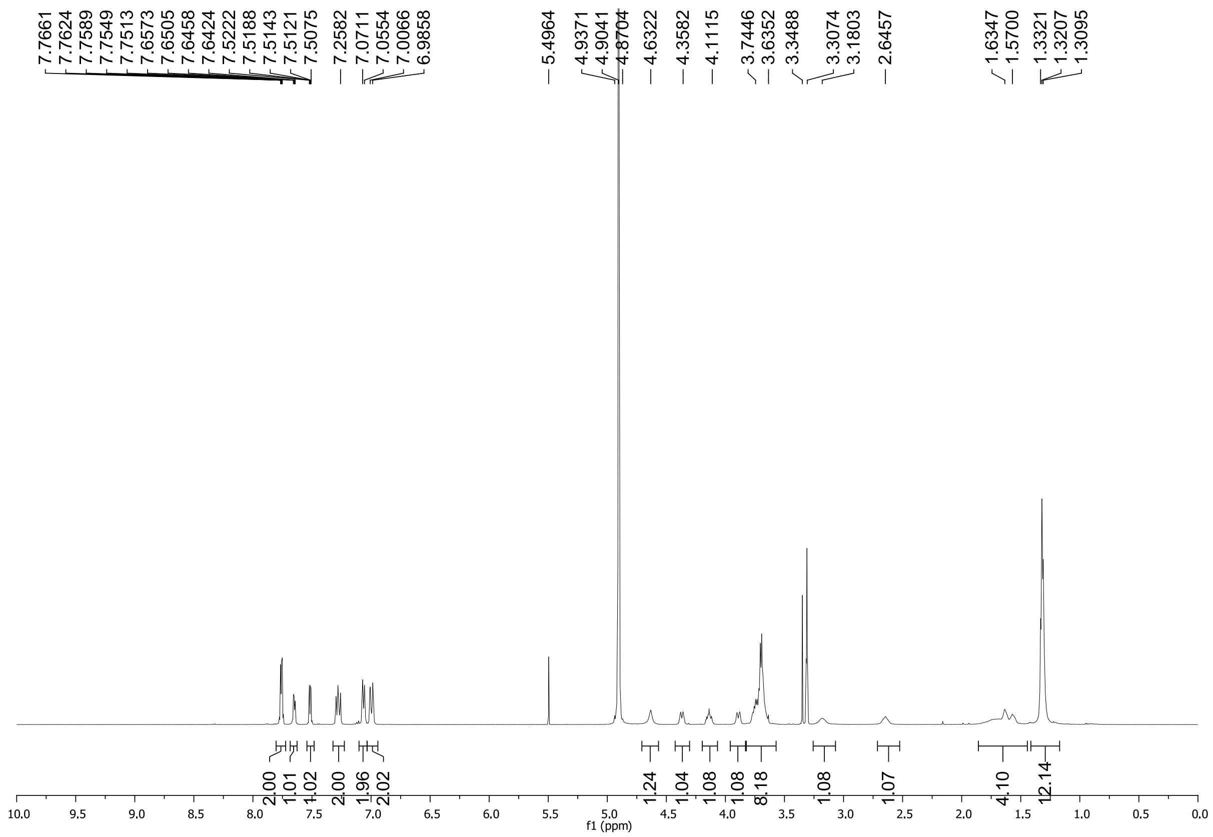

4. Cloning and Expression of GFP and GFP-SH

The gene for superfolder GFP gene was kindly donated by the Registry of Standard Biological Parts. This was cloned into the pNIC28-Bsa4 vector using the primers TACTTCCAATCCATGCGTAAAGGCGAAGAGCTGTTCAC and TATCCACCTTTACTGTTATTTGTATAGTTCATCCATACCATGCGTG. GFP-SH was derived from superfolder GFP by site directed mutagenesis, using the primers CATAAGCTGGAATACAATTTTAACTGCCACAATGTTTACATCACCGCC and GGCGGTGATGTAAACATTGTGGCAGTTAAAATTGTATTCCAGCTTATG. An inconsequential E→D mutation occurred in the N-terminal linker between the His_6 tag and the GFP domain.

Cloned Superfolder GFP sequence
MHHHHHHSSGVDLGT**E**NLYFQSMRKGEELFTGVVPILVELDGDVNGHKFSV
RGEGEGDATNGKLTLKFICTTGKLPVPWPTLVTTLTYGVQCFARYPDHMKQ
HDFFKSAMPEGYVQERTISFKDDGTYKTRAEVKFEGDTLVNRIELKGIDFKED
GNILGHKLEYNFN**S**HNVYITADKQKNGIKANFKIRHNVEDGSVQLADHYQQ
NTPIGDGPVLLPDNHYLSTQSVLSKDPNEKRDHMVLLEFVTAAGITHGMDEL
YK

GFP-SH sequence
MHHHHHHSSGVDLGT**D**NLYFQSMRKGEELFTGVVPILVELDGDVNGHKFSV
RGEGEGDATNGKLTLKFICTTGKLPVPWPTLVTTLTYGVQCFARYPDHMKQ
HDFFKSAMPEGYVQERTISFKDDGTYKTRAEVKFEGDTLVNRIELKGIDFKED
GNILGHKLEYNFN**C**HNVYITADKQKNGIKANFKIRHNVEDGSVQLADHYQQ
NTPIGDGPVLLPDNHYLSTQSVLSKDPNEKRDHMVLLEFVTAAGITHGMDEL
YK

The vector was transformed into BL21(DE3)plysS cells, and proteins were expressed at 37 ^{0}C using standard methods. Proteins were purified by nickel chromatography, and the buffer was exchanged for 100 mM sodium phosphate pH 8 by repeated diafiltration at 4^{0}C. Purity of the proteins was confirmed by SDS-PAGE (figure S1).

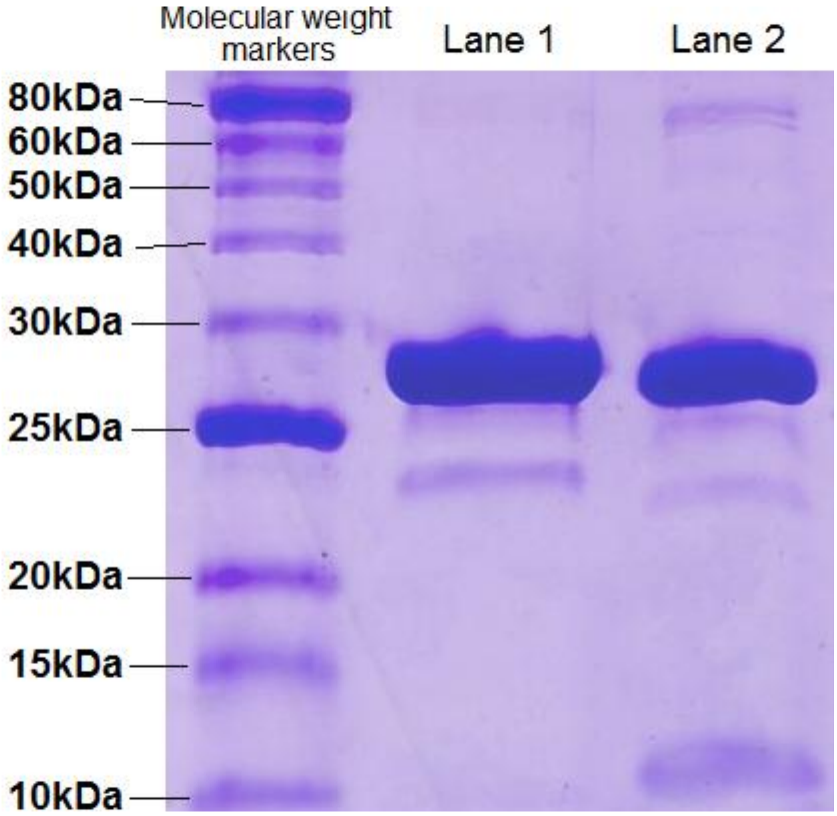

Figure S1. SDS-PAGE gel of Superfolder GFP (Lane 1) and GFP-SH (Lane 2).

Reactivity of GFP with *N*-Methyl-monobromomaleimide

N-methyl-monobromomaleimide (15 mM in DMF, 4 μL) was added to wild type superfolder GFP (33 μM, 196 μL) in 100 mM sodium phosphate buffer pH 8. This was incubated for 2 hours at room temperature. Under these conditions, no significant change in the mass of GFP was observed, suggesting that C48 and C70 of the wild type protein are unreactive towards bromomaleimides.

A)

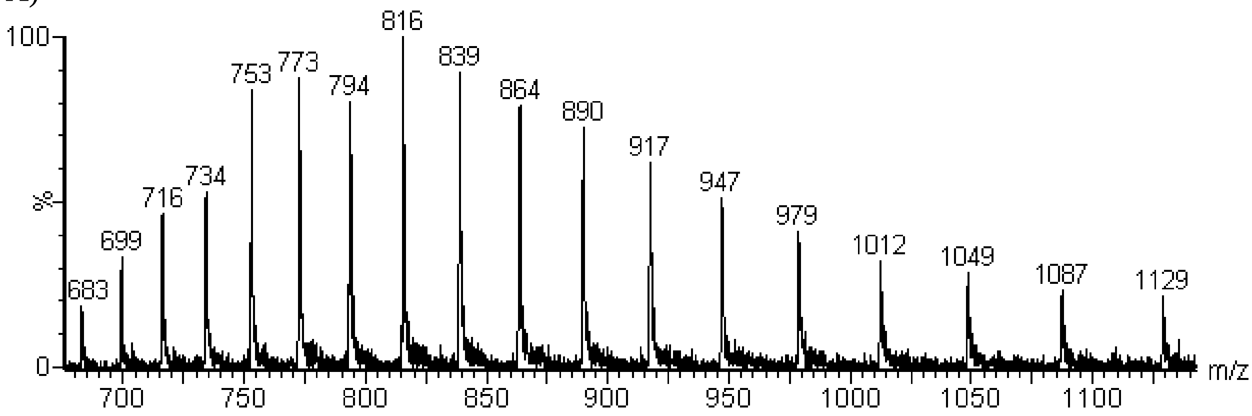

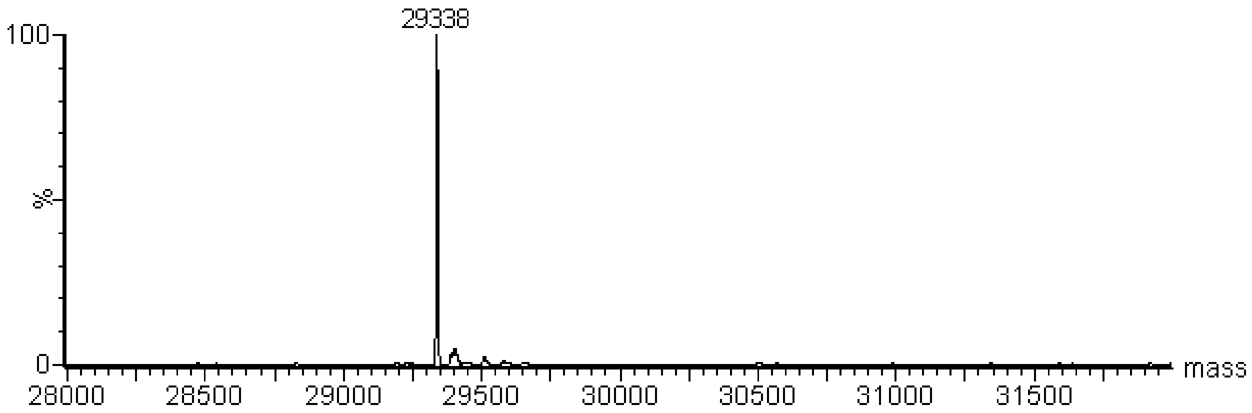

B)

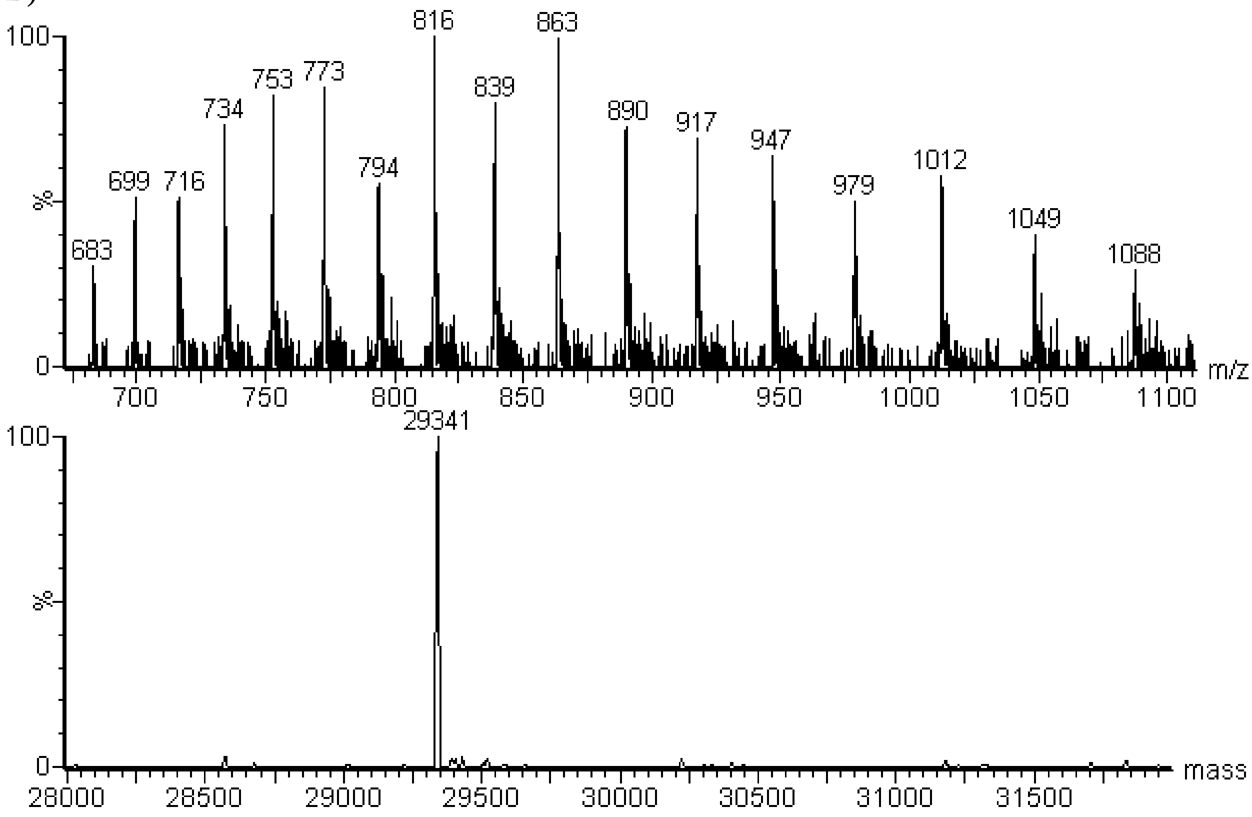

Sub-figure	Compound formed	Expected mass	Observed mass
A	Superfolder GFP	29341	29338
B	Superfolder GFP	29341	29341

Figure S2. Raw and Deconvoluted Mass Spectra of Wild Type Superfolder GFP. The expected mass of superfolder GFP was calculated by the Expasy Protparam webserver, minus 18Da due to maturation of the chromophore[2]
(A) Unmodified. (B) After incubation with *N*-methyl-monobromomaleimide.

5. Assembly of GFP-SH / Rhodamine FRET Pairs

Derivatisation reactions were performed in 100 mM pH 8 sodium phosphate buffer on ice for 1 h. A stock solution of maleimide was made up in dimethylformamide (DMF). To form conjugates **7-9**, a 10-fold molar excess of compounds **4-6** in DMF was added to a buffered solution of GFP-SH. For formation of compound **10**, one equivalent of compound **6** in DMF was added to two equivalents of buffered GFP-SH. Formation of the expected products were confirmed by electrospray mass spectrometry (Figure S3). Removal of excess rhodamine reagent was performed by repeated diafiltration with 100 mM pH 8 sodium phosphate buffer at 4 ^{0}C.

A)

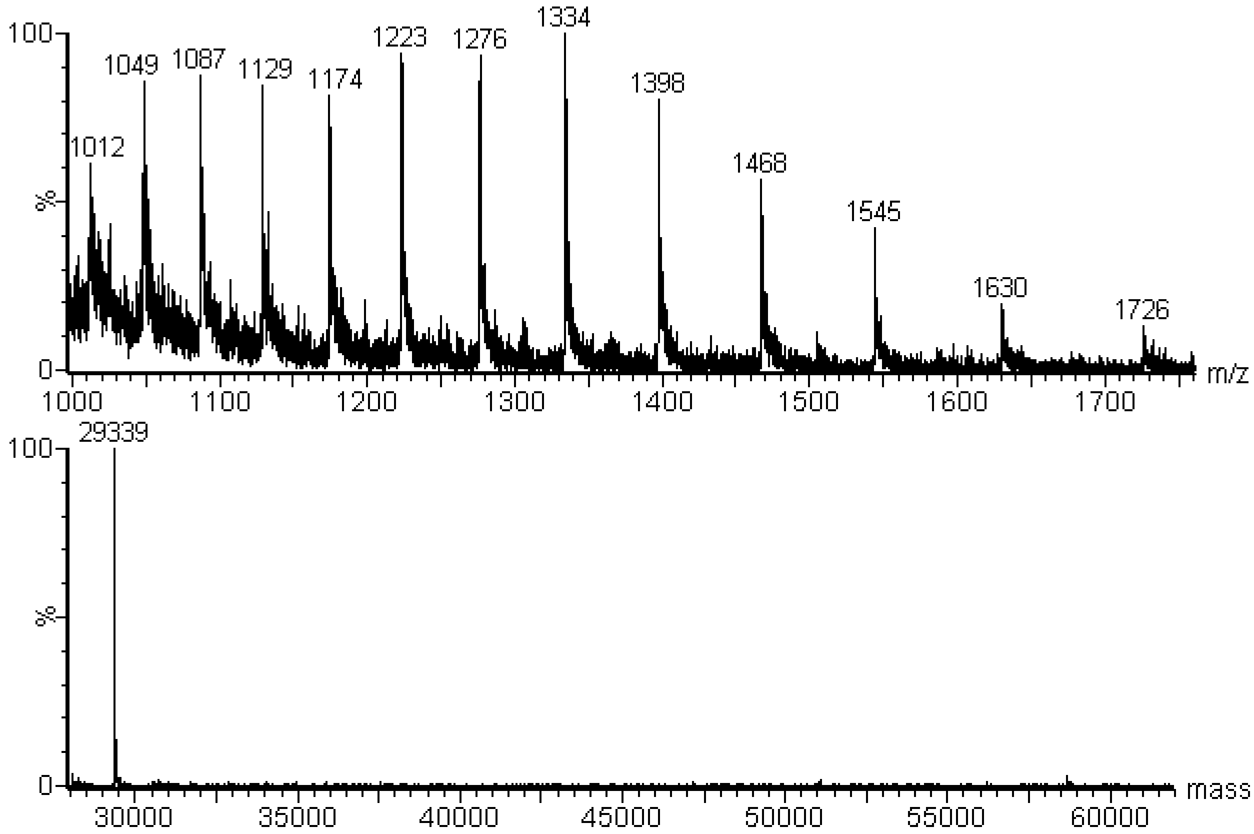

B)

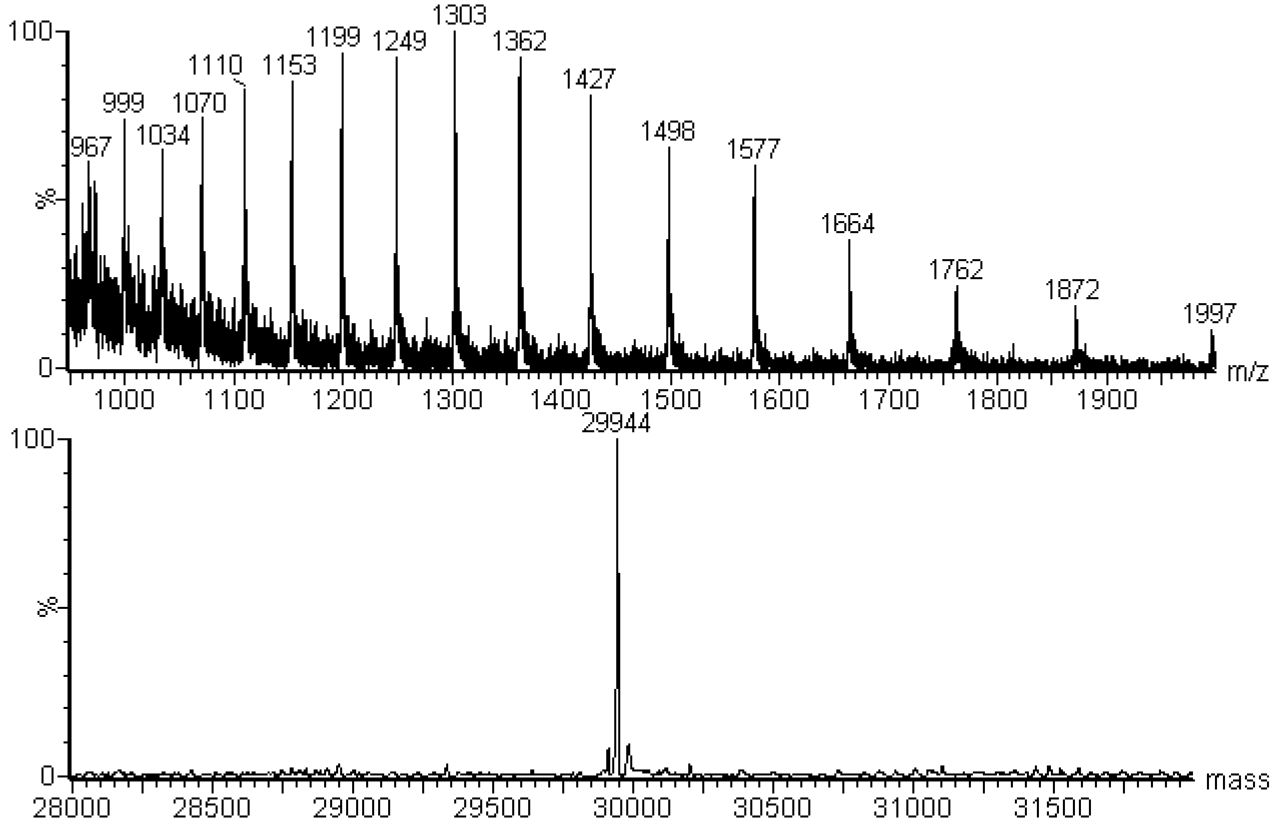

967
999
1034
1070
1110
1153
1199
1249
1303
1362
1427
1498
1577
1664
1762
1872
1997
100
%
0
m/z
1000
1100
1200
1300
1400
1500
1600
1700
1800
1900
29944
100
%
0
mass
28000
28500
29000
29500
30000
30500
31000
31500

C)

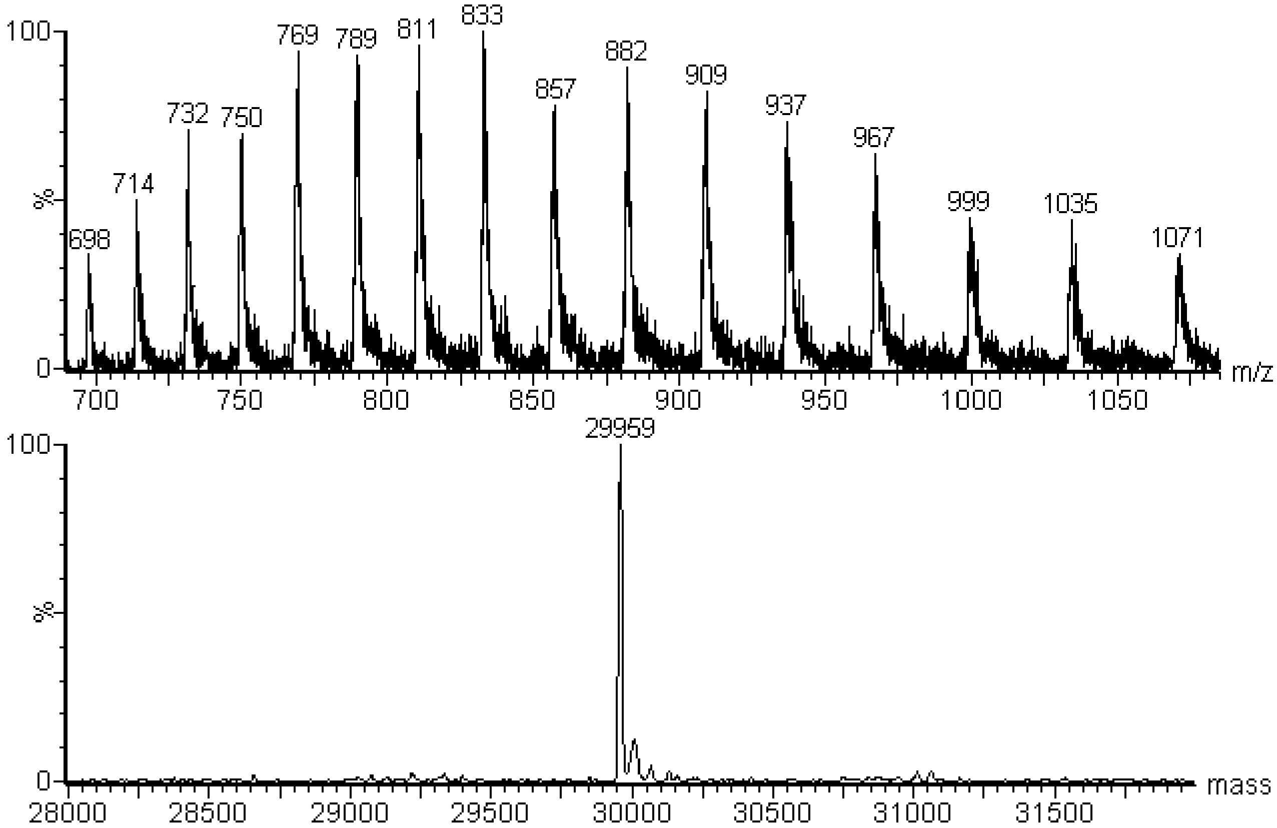

698
714
732
750
769
789
811
833
857
882
909
937
967
999
1035
1071
100
%
0
m/z
700
750
800
850
900
950
1000
1050
29959
100
%
0
mass
28000
28500
29000
29500
30000
30500
31000
31500

D)

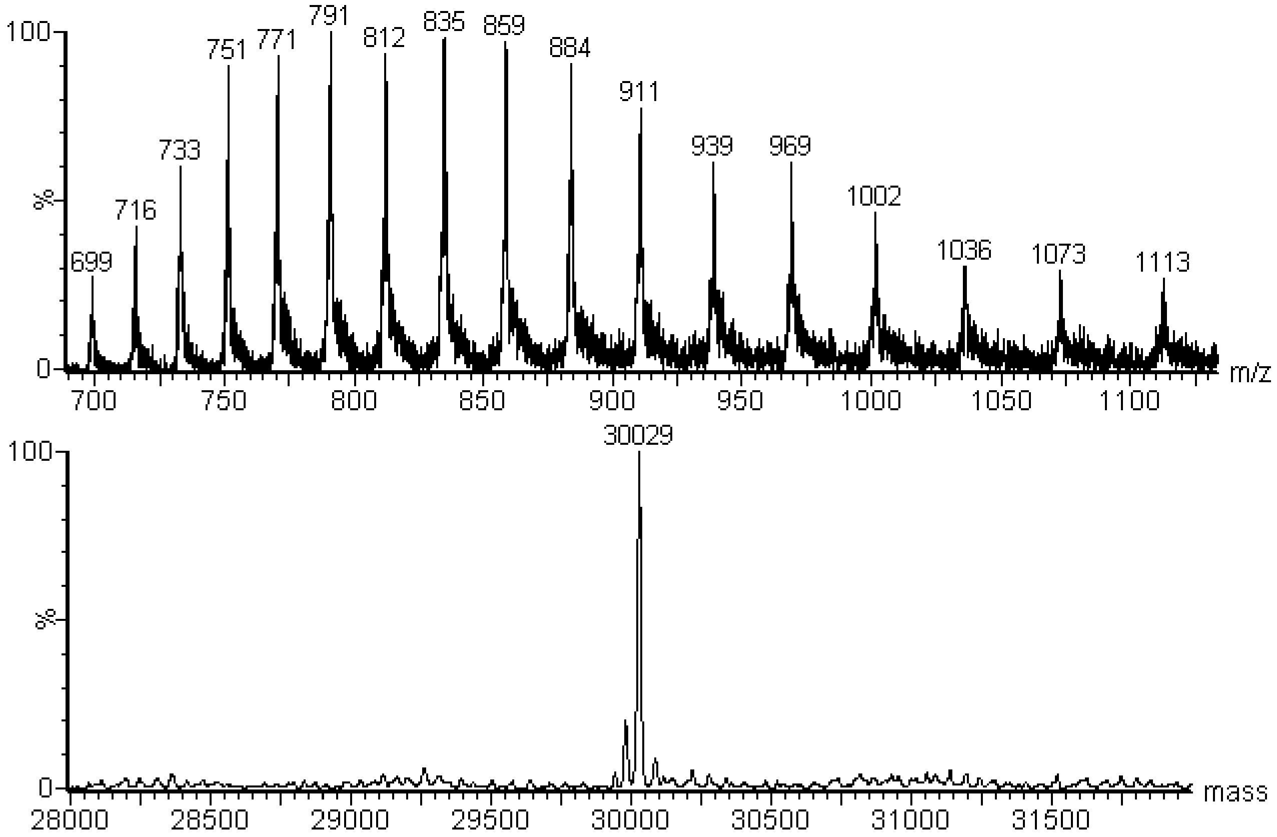

699
716
733
751
771
791
812
835
859
884
911
939
969
1002
1036
1073
1113
100
%
0
700 750 800 850 900 950 1000 1050 1100
m/z
30029
100
%
0
28000 28500 29000 29500 30000 30500 31000 31500
mass

E)

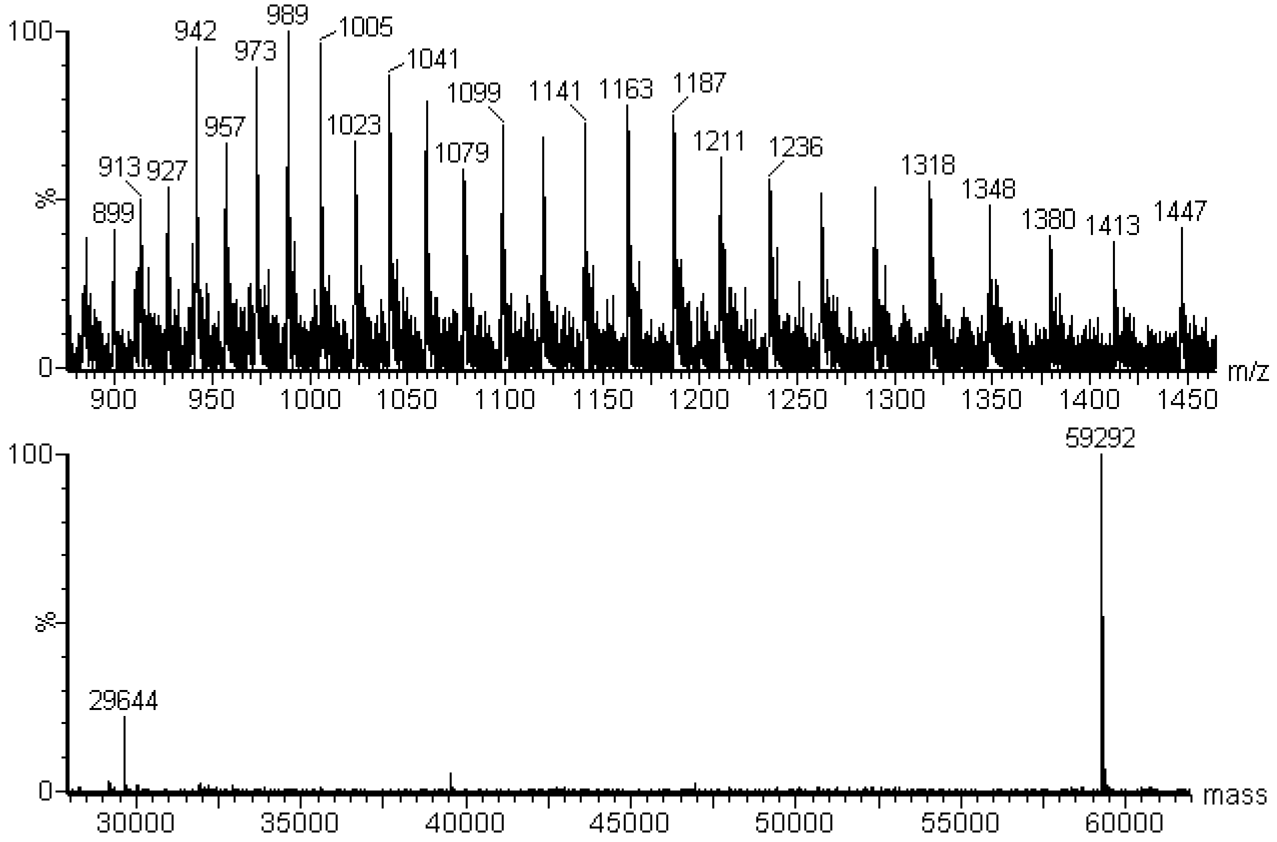

899
913
927
942
957
973
989
1005
1023
1041
1079
1099
1141
1163
1187
1211
1236
1318
1348
1380
1413
1447
100
%
0
900 950 1000 1050 1100 1150 1200 1250 1300 1350 1400 1450
m/z
59292
29644
100
%
0
30000 35000 40000 45000 50000 55000 60000
mass

Sub-figure	Compound formed	Expected mass	Observed mass (Da)
A	GFP-SH	29343	29338
B	Compound 7	29948	29944
C	Compound 8	29946	29959
D	Compound 9	30026	30029
E	Compound 10	59292	59292

Figure S3. Mass Spectrometry of GFP/Rhodamine FRET pairs.
The expected mass of superfolder GFP was calculated by Expasy Protparam, minus 18Da due to maturation of the chromophore[1].
(A) GFP-SH. (B) Compound **7**. (C) Compound **8**. (D) Compound **9**. (E) Compound **10**.

6. Determination of Optical Properties

Absorbance spectra were obtained using a Cary 100 bio UV-visible spectrophotometer using a 10µM solution of fluorescent compound, in PBS pH 7.4 at room temperature. Fluorescence properties were measured using a Cary Eclipse fluorescence spectrophotometer using a 1µM of fluorescent compound, in PBS pH 7.4 at 20ºC.

6.1 Rhodamine Compounds 4-6

A)

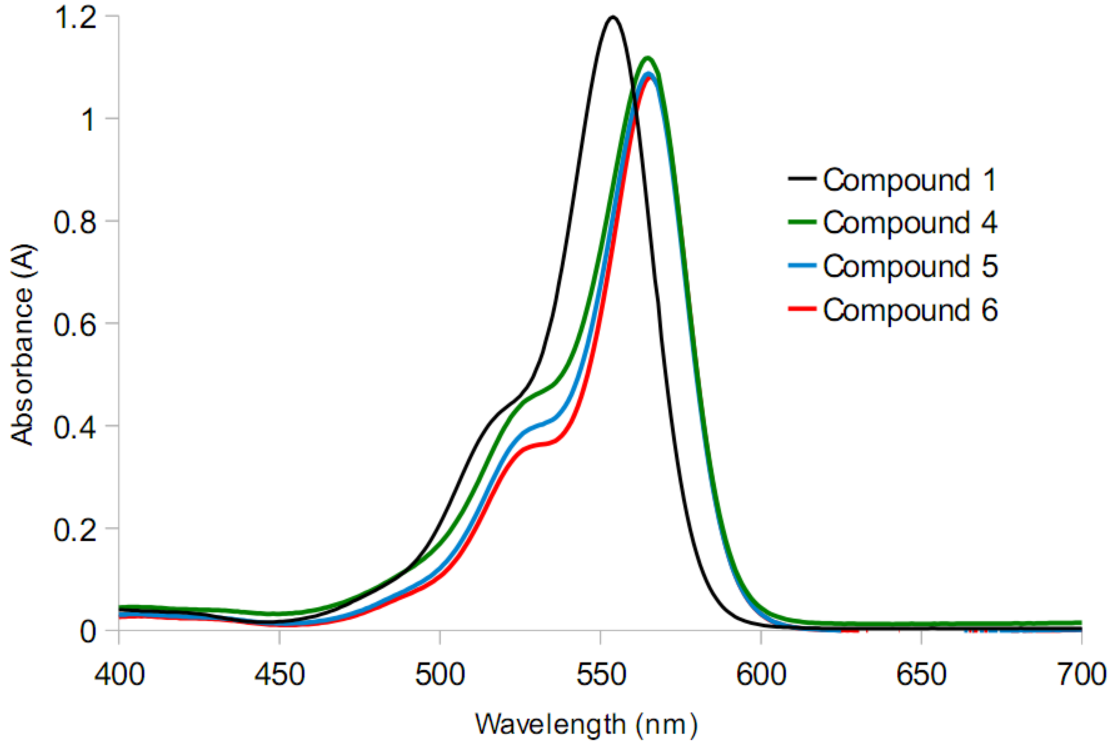

B)

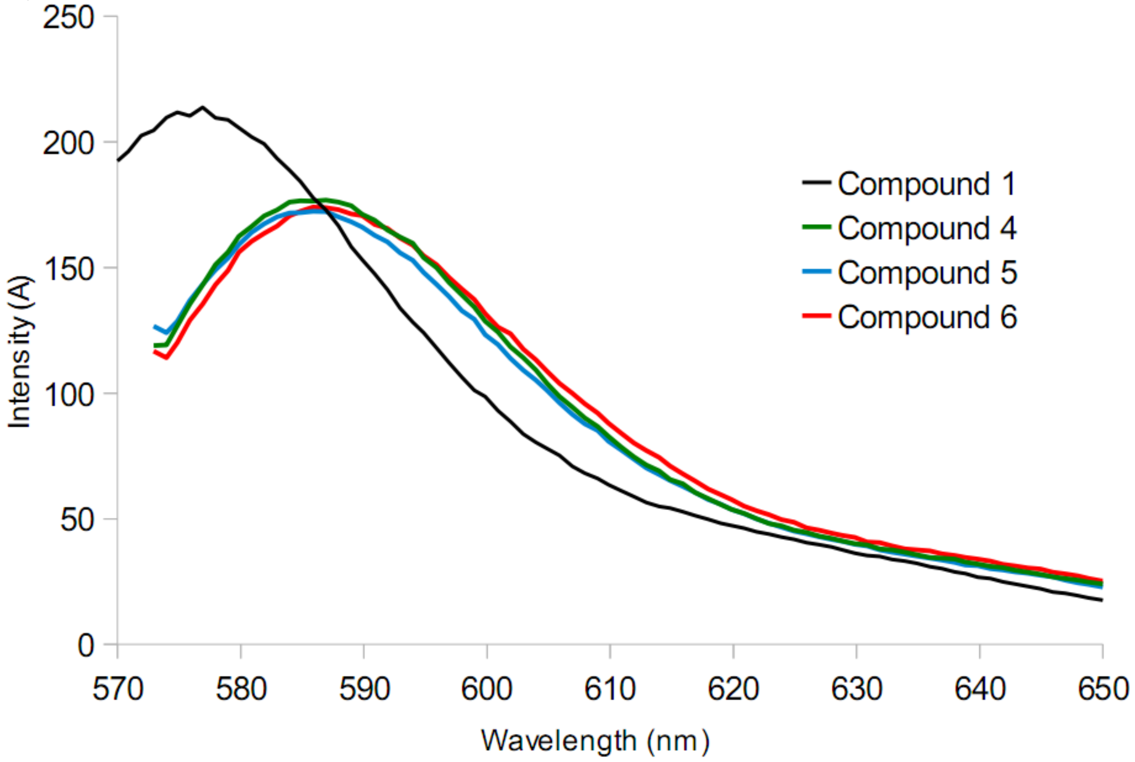

Figure S4. Optical Characteristics of Compounds **4-6** in PBS at Room Temperature. (A) Absorbance spectra. (B) emission spectra: Compound **1** is excited at 555nm, compounds **4-6** are excited at 565nm.

6.2 GFP Constructs 7-10

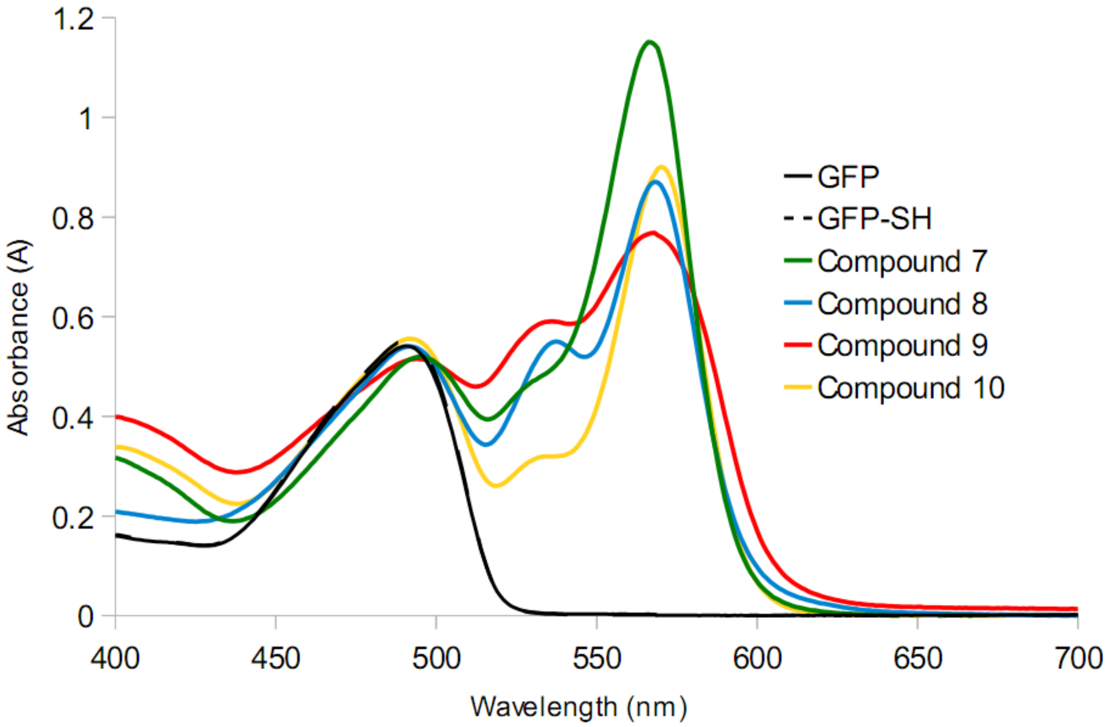

Figure S5. Absorbance Spectra of 100μM of Compounds **7-10** in PBS at Room Temperature.

7 In vitro Cleavage Studies for Conjugates 7-10

A solution of construct (0.85 μM for **7-9**, 0.43 μM for **10**) was made up in PBS buffer (pH 7.4, 100 μL) and the solution maintained at 20°C . The intensities for the following excitation and emission wavelengths were measured alternately:

Excitation at 494 nm, emission at 514 nm (GFP).
Excitation at 494 nm, emission at 590 nm (rhodamine).

After a short pre-record time, 2 mM reduced glutathione (100 μL) was added and rapidly mixed by repeated pipette aspiration and ejection and the progress of the reaction followed photometrically. The pre-record intensities were later halved to correct for dilution.

Cleavage of each compound was confirmed by electrospray mass spectrometry. Constructs (34 μM) were incubated with 1 mM reduced glutathione for 1 h at room temperature.

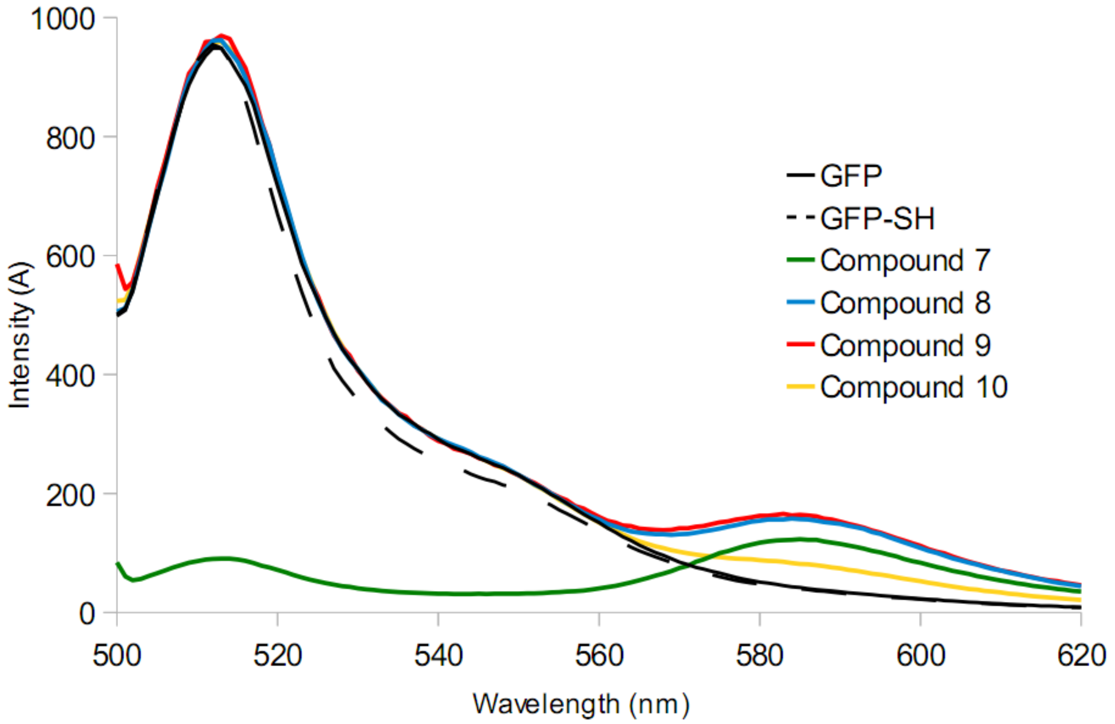

Figure S6. Emission Spectra after Cleavage of Constructs **7-10**.
Excitation at 494nm.

A)

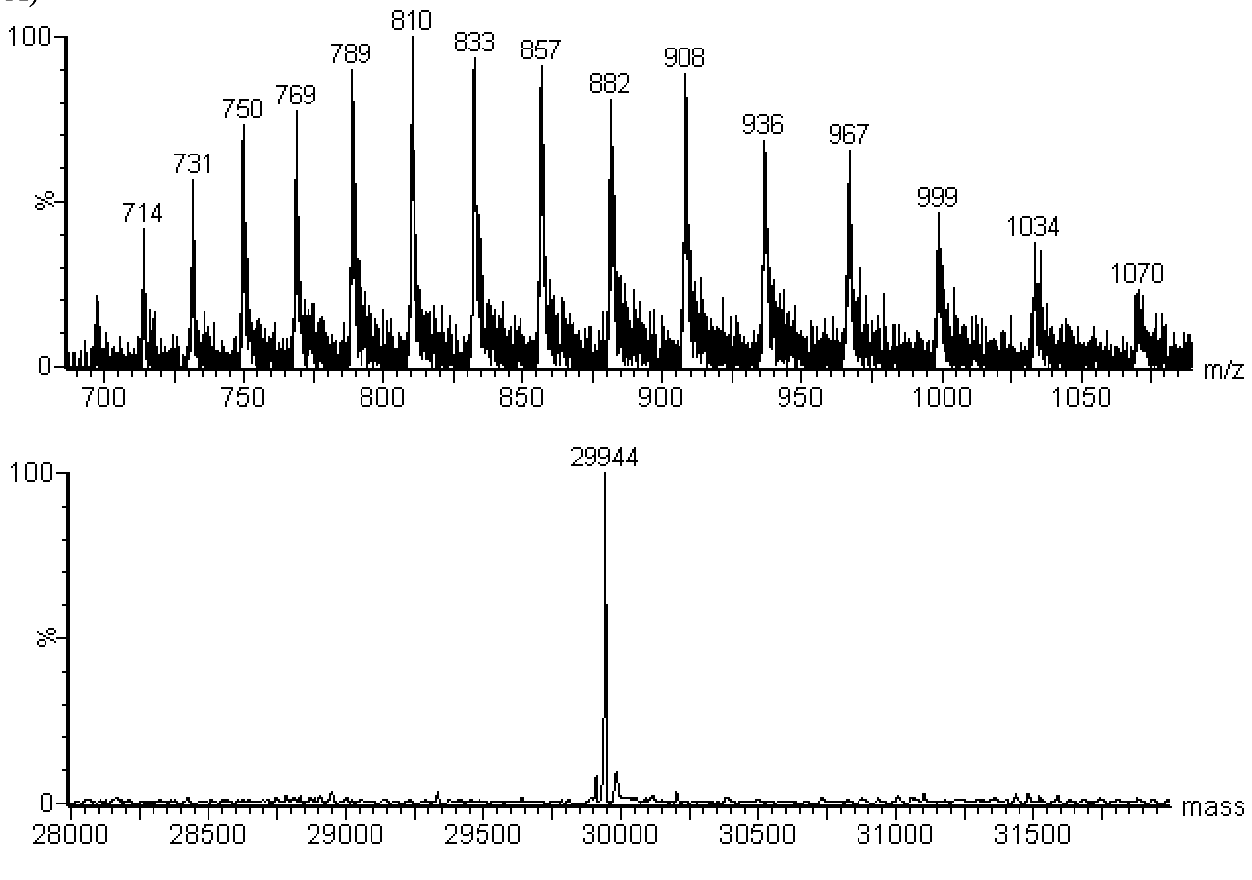

B)

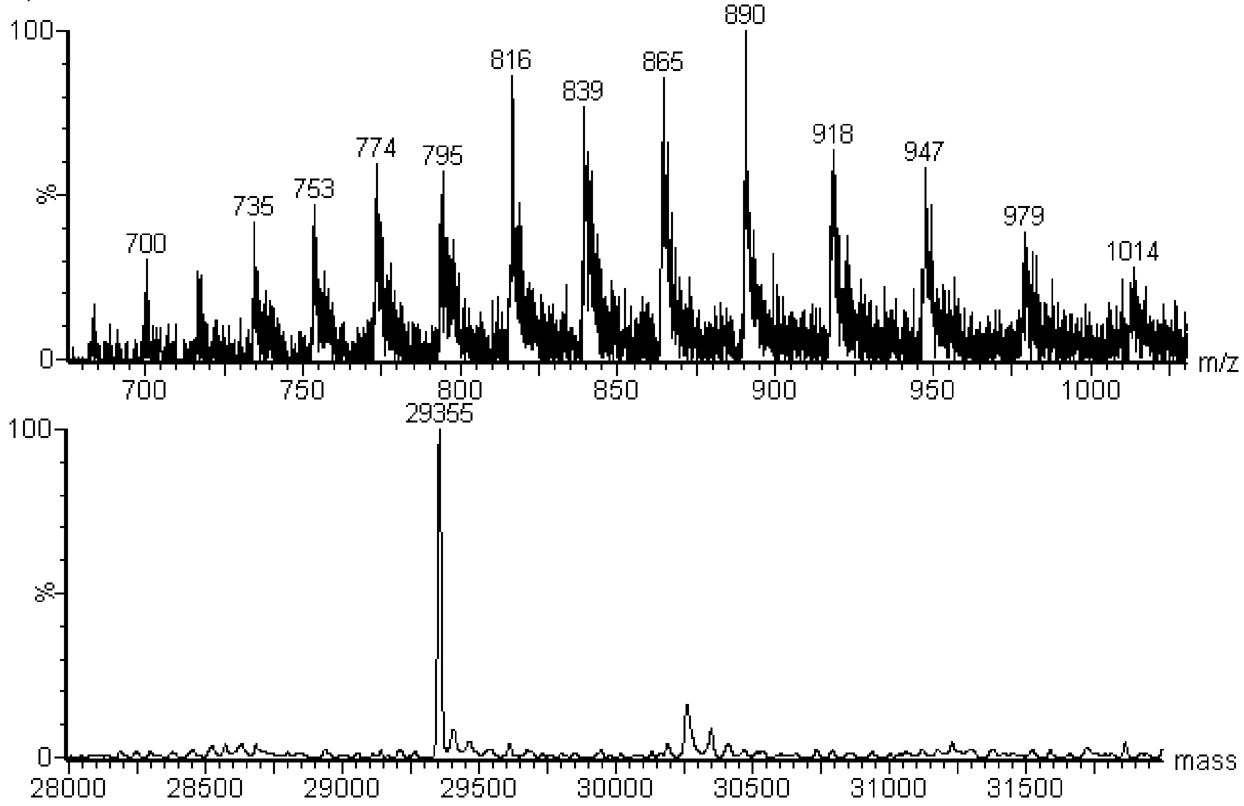

100
%
0
700
735
753
774
795
816
839
865
890
918
947
979
1014
700
750
800
850
900
950
1000
m/z
29355
100
%
0
28000
28500
29000
29500
30000
30500
31000
31500
mass

C)

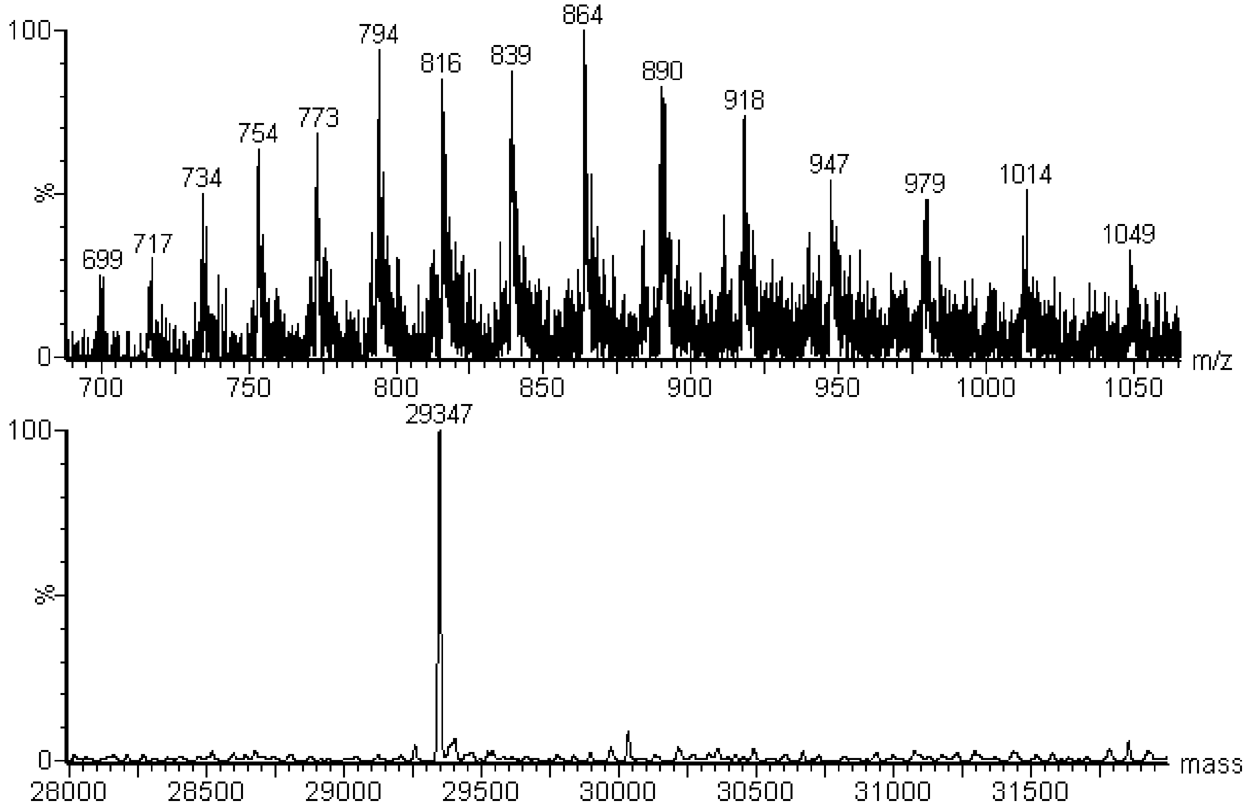

100
%
0
699
717
734
754
773
794
816
839
864
890
918
947
979
1014
1049
700
750
800
850
900
950
1000
1050
m/z
29347
100
%
0
28000
28500
29000
29500
30000
30500
31000
31500
mass

D)

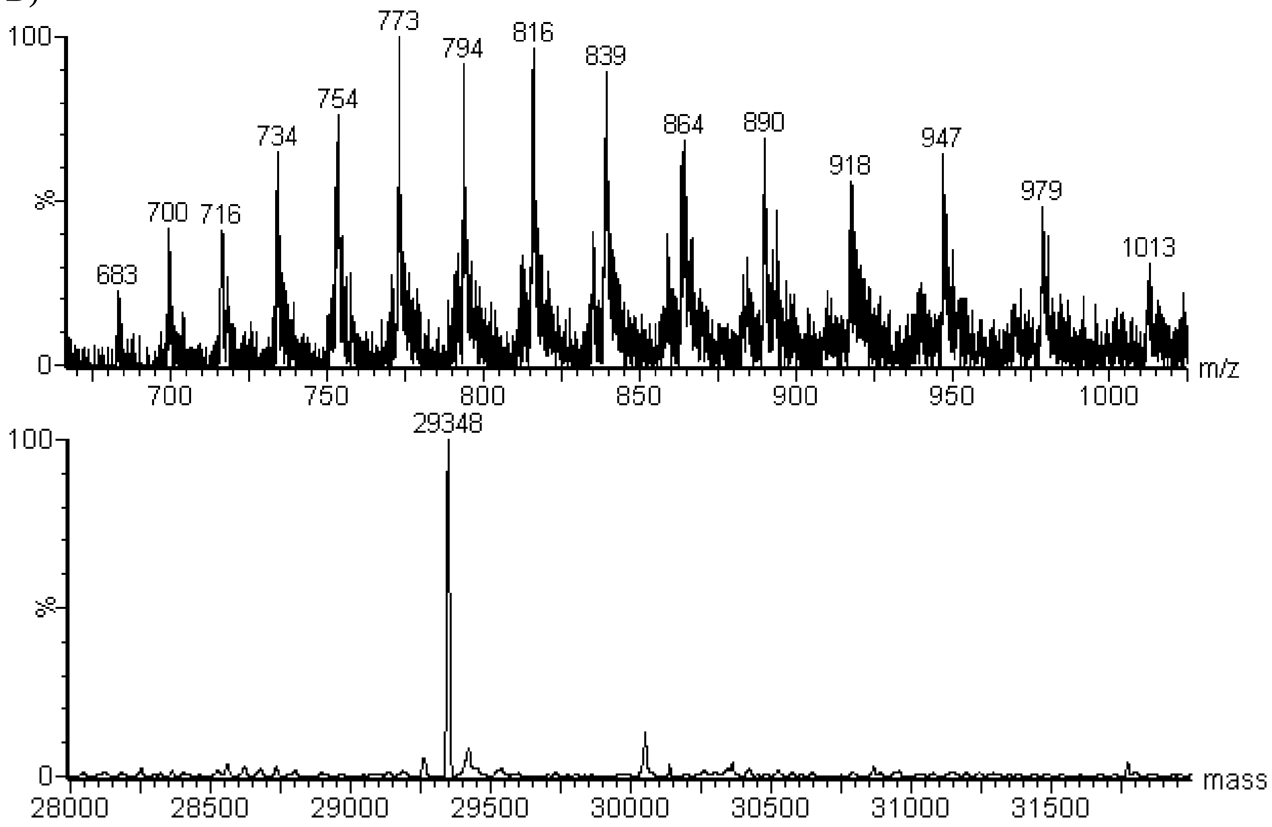

Sub-figure	Compound formed	Expected mass	Observed mass (Da)
A	Compound 7	29948	29944
B	GFP-SH	29343	29355
C	GFP-SH	29343	29347
D	GFP-SH	29343	29348

Figure S7. Electrospray Mass Spectra of Constructs **7-10** Following Treatment with Glutathione.
(A) Compound 7 (B) Compound 8 (C) Compound 9 (D) Compound 10.

8 In cell Cleavage Studies for Conjugates 7-10

8.1 Cell culture for Microinjection Experiments

HeLa and COS-7 cells were cultured using DMEM media with 10% bovine calf serum, 100 µg/ml streptomycin and 100 units/ml penicillin in 20ml Nunclon flasks. These were stored in a 37ºC incubator with 5% $CO_{2.}$ Cells were removed from the culture tray using trypsin and added to coverslips in a 6 well plate (nunclon), to a cell

density that resulted in roughly 20% confluence. The cells were left overnight at 37ºC with 5% CO_2 to allow adherence of the cells to the coverslip.

The coverslips were assembled into the base of a custom-designed chamber, kindly donated by Dr. Gregory Mashanov. A perfusion system was set up to ensure a constant flow of Hanks buffer over the cells and the microinjection tip (figure S8).

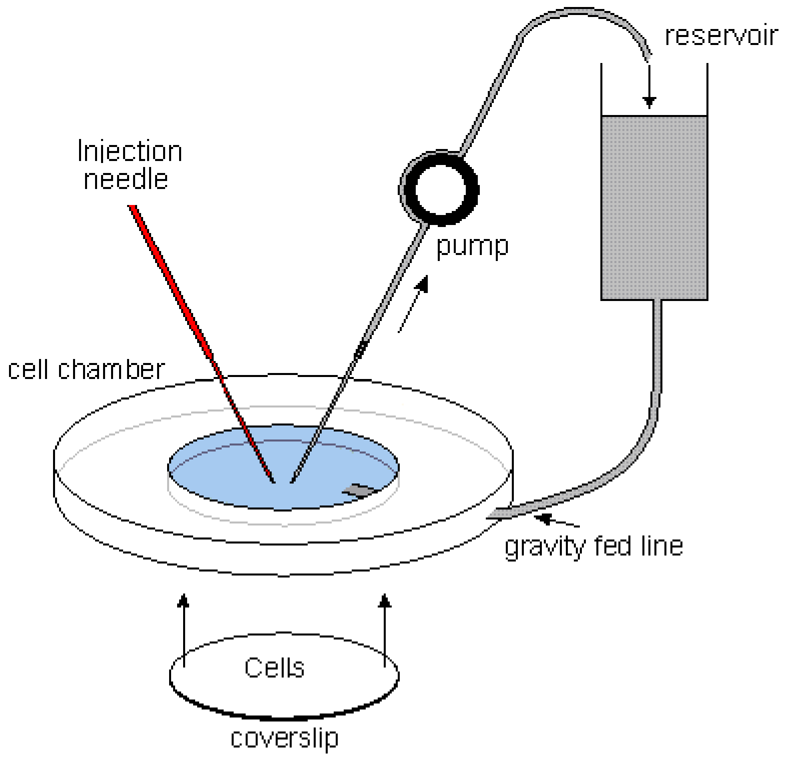

Figure S8. Perfusion System for Microinjection.

8.3 Microinjection

Microinjection was performed using Transjector 5246 (Eppendorf) and an InjectMan (Eppendorf) injection systems, kindly donated by Dr. Tom Carter. Borosilicate glass capillaries with internal filaments (GC100TF-10, Warner instruments) were pulled using a Narishige PD-5 pipette puller.

FRET constructs **7-10** were diluted to 3.4 μM concentration in PBS (pH 7.4) and filtered through a 0.2μm filter. The filtered solutions were backfilled into the capillaries. For injection into cells, the backpressure was set to 10 hPa and the injection time was set to 0.5 s. The injection pressure was set to between 120 hPa and 200 hPa for HeLa cells and 60 hPa and 110 hPa for COS-7 cells.

8.4 Microscopy

Both differential interference contrast (DIC) and fluorescence images were recorded using a Nikon diaphot microscope,with a 20xDIC objective (0.4 NA), a Nikon widefield zoom magnification module set to 1x magnification and an Dual-View image splitter (Optical Insights) in series. The image splitter contained a 565dcxr dichroic mirror, a Q525/50m filter for GFP emission, and a HQ605/55m filter for rhodamine emission. A CCD camera (WAT-902H, Watec) with fixed gain was used for image detection.

Fluorescence imaging was performed using the excitation filter and dichroic mirror from a 49002 eGFP filter cube (Chroma).

A cube holder allowed rapid manual switching between a Wollaston prism (for differential interference contrast (DIC) imaging) and the eGFP filter cube (for fluorescence imaging). A HB-10101AF mercury lamp was used for fluorescence imaging, and a halogen lamp was used for DIC imaging. The injection process was recorded using DIC optics, and then rapidly changed to fluorescence imaging.

8.5 Analysis of Microinjection Data

All videos were analysed using imageJ.[3] Identical regions of interest were selected in the rhodamine and GFP channels for the cell and for background. The average intensity for these regions were calculated for each frame. Videos taken using the opti-split were analysed per frame, and the GFP/FRET ratio was calculated as:

$$\frac{(Average\, GFP intensity\, of\, cell) - (Average\, GFP\, intensity\, of\, background)}{(Average\, rhodamine\, intensity\, of\, cell) - (Average\, rhodamine\, intensity\, of\, background)}$$

An exponential model was fit to the data by a least differences fit using the Microsoft Excel 2003 solver function.

8.6 Raw GFP and Rhodamine Emission Data

Representative traces for each compound are shown in figure S9.

A)

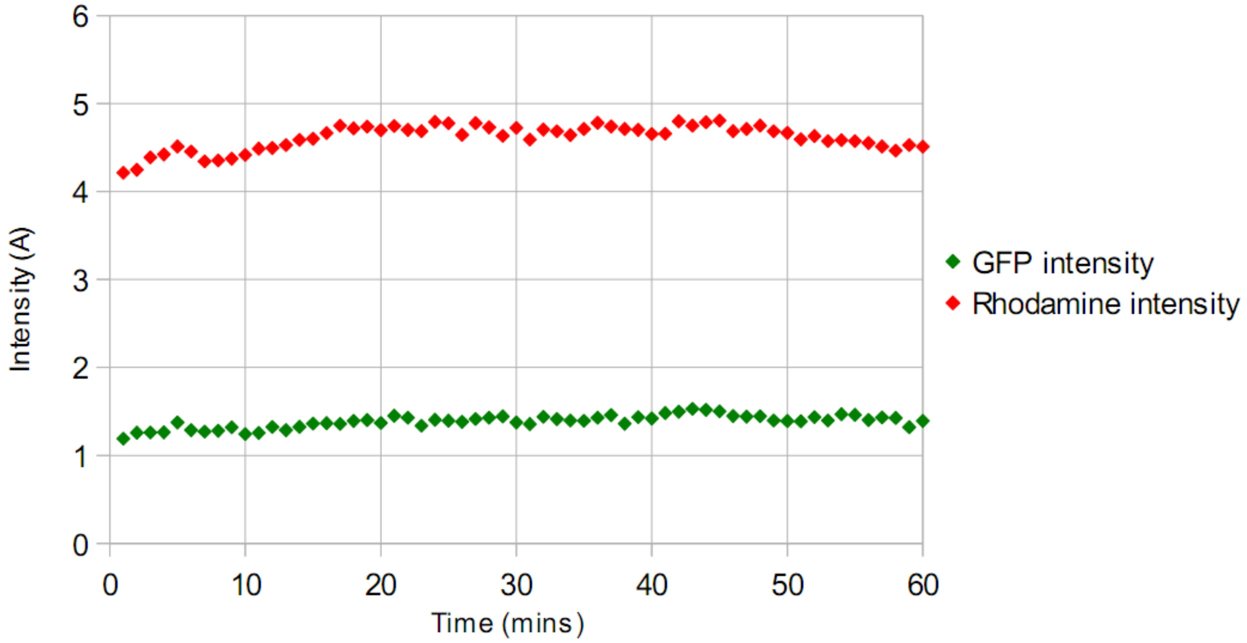

B)

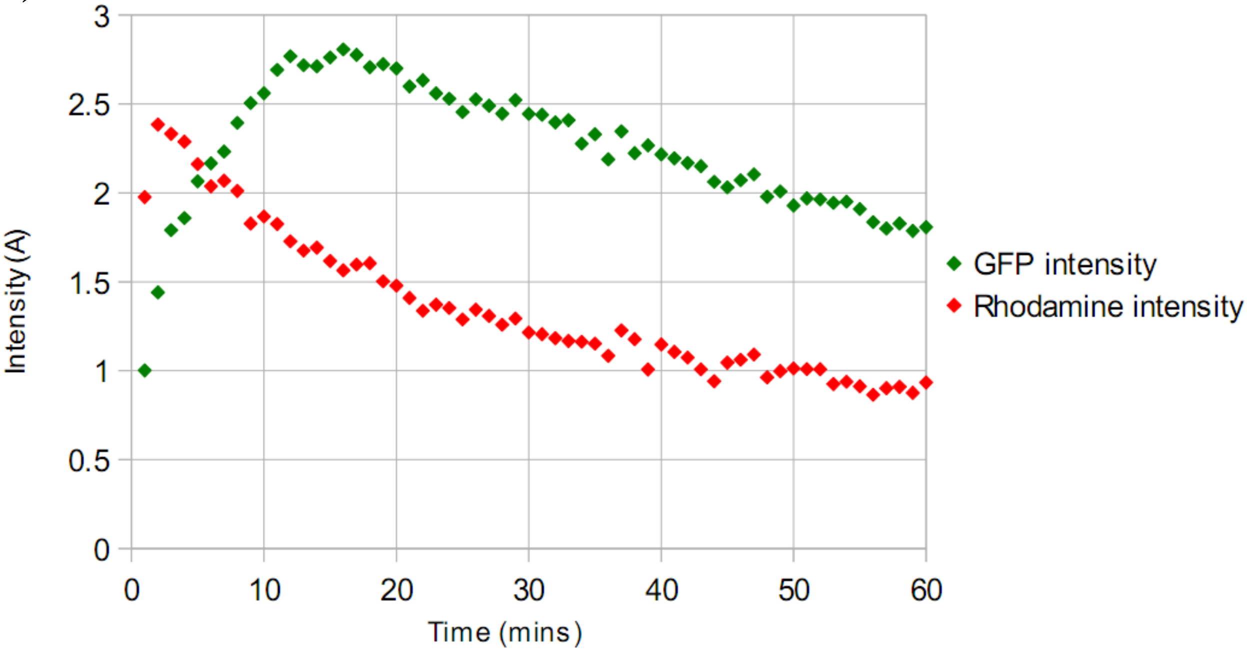

C)

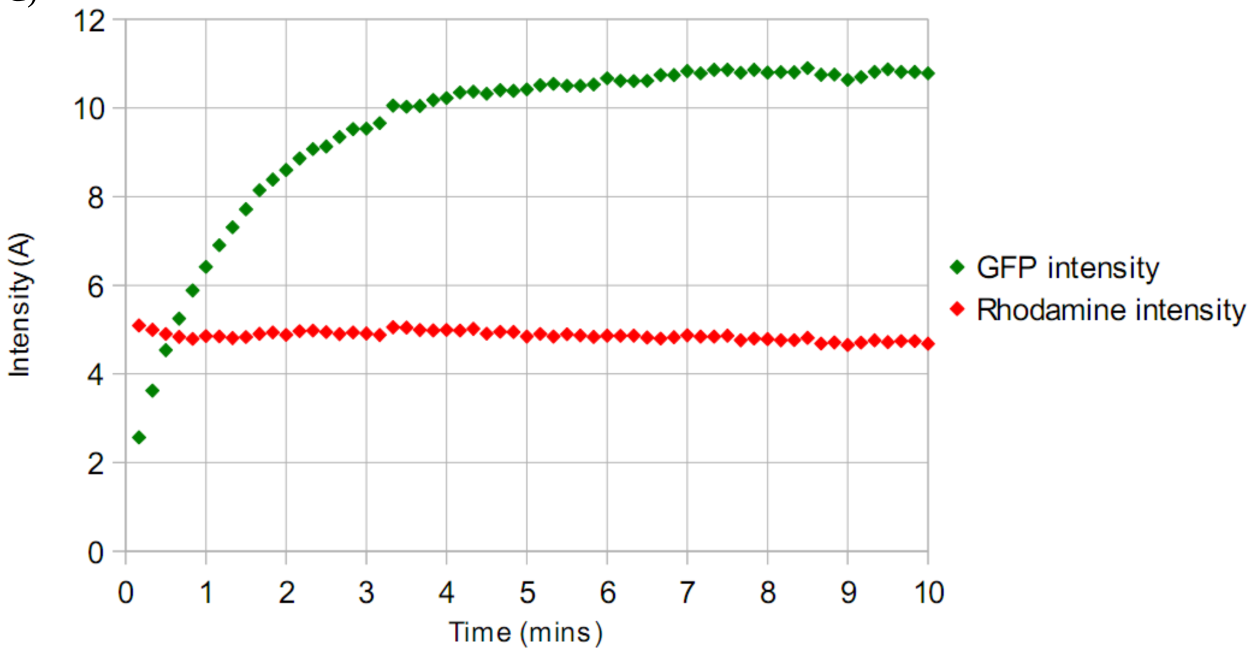

12
10
8
6
4
2
0
Intensity (A)
0
1
2
3
4
5
6
7
8
9
10
Time (mins)
GFP intensity
Rhodamine intensity

D)

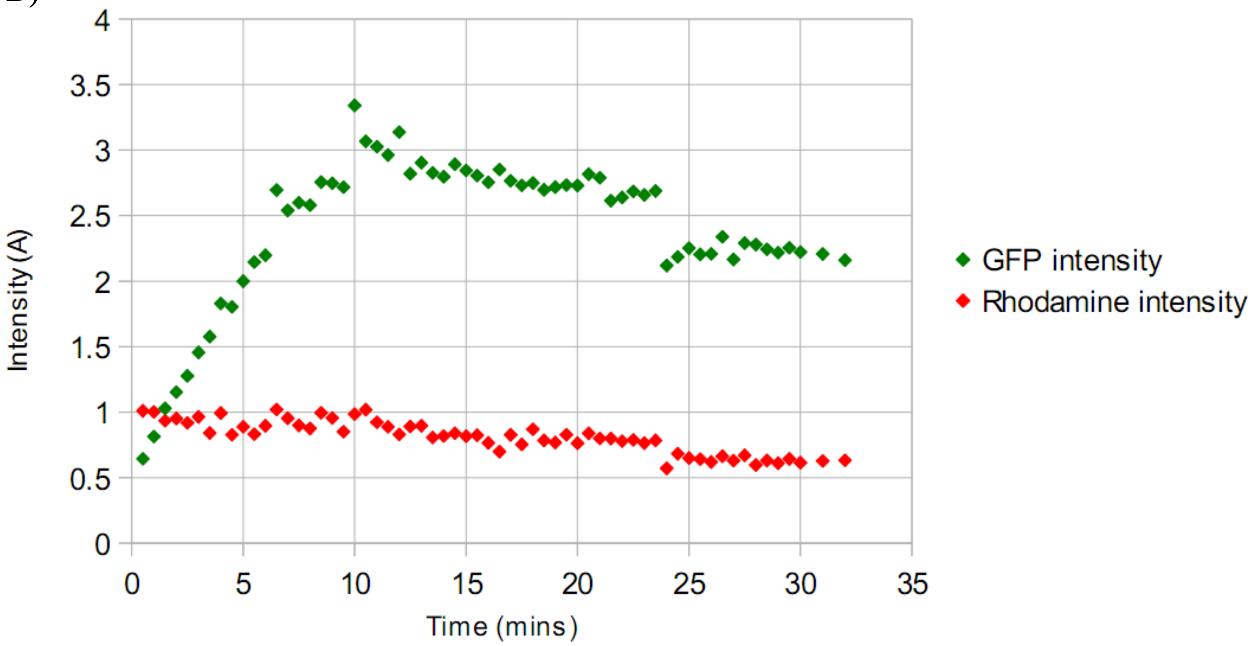

4
3.5
3
2.5
2
1.5
1
0.5
0
Intensity (A)
0
5
10
15
20
25
30
35
Time (mins)
GFP intensity
Rhodamine intensity

E)

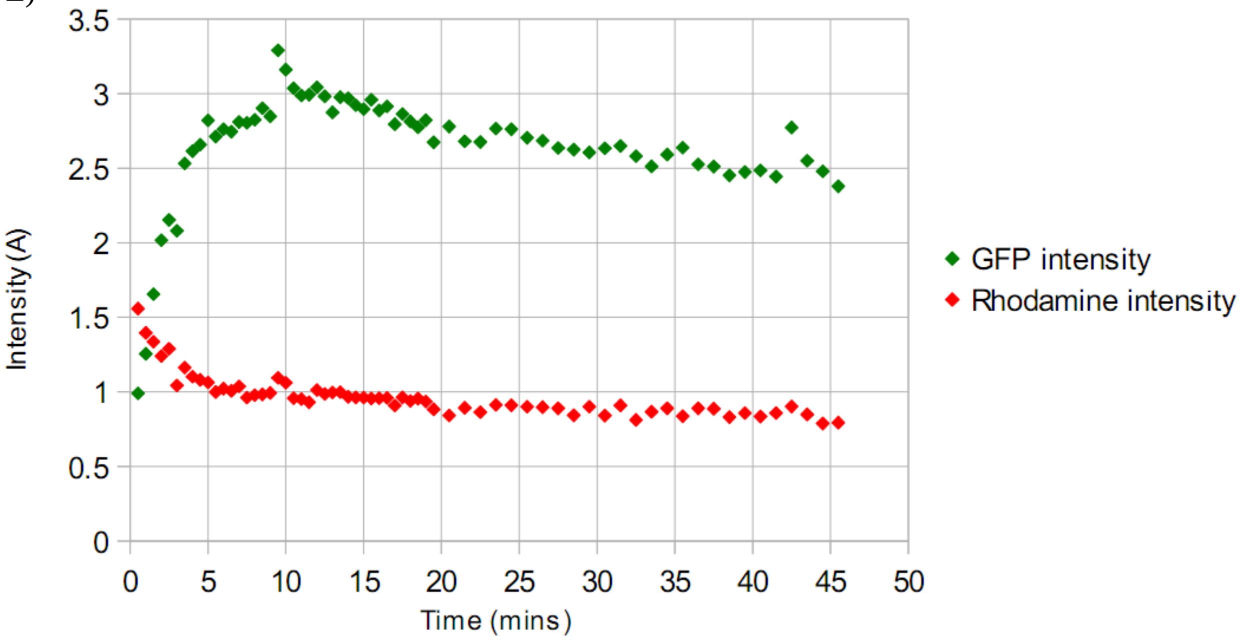

Figure S9. Raw Data for Emission Intensities of GFP and Rhodamine Following Microinjection of (A) Compound **7** into HeLa cells. (B) Compound **8** into HeLa cells. (C) Compound **9** into HeLa cells. (D) Compound **10** into HeLa cells. (E) Compound **10** into COS-7 cells.

9 Comparison of Kinetics in vitro and in cell

A comparison of the rates of reaction observed, derived from GFP/rhodamine emission ratio profiles, is shown for the in vitro fluorimetry and in cell microinjections experiments in Figure S10.

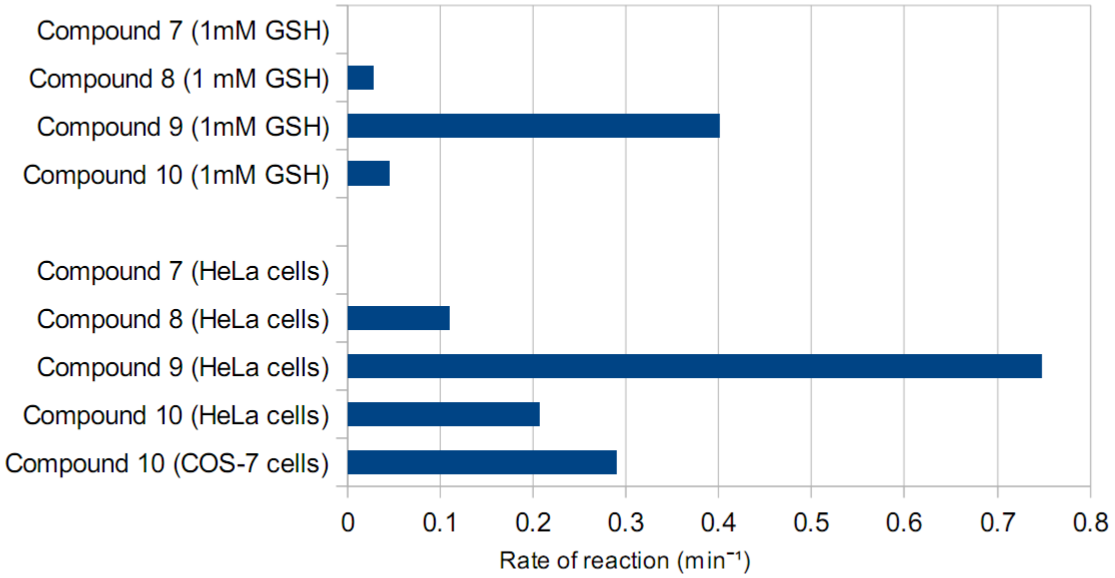

Figure S10. Rates of Reaction Observed for Constructs **7-10** Obtained by Fluorimetry and by Microinjection.
(The values for microinjection experiments are an average from two injections.)

10 Measuring the Fluorescence of Compounds Cleaved in vitro Using Fluorescence Microscopy

Solutions of constructs **7-10** (3.4 μM) were made up with 10mM glutathione in PBS at pH 7.4. Small droplets were spotted onto a glass bottom culture dish (MatTek), and immediately covered in silicone oil. The relative emission intensities of GFP and rhodamine were then calculated as described in Section 8.5.

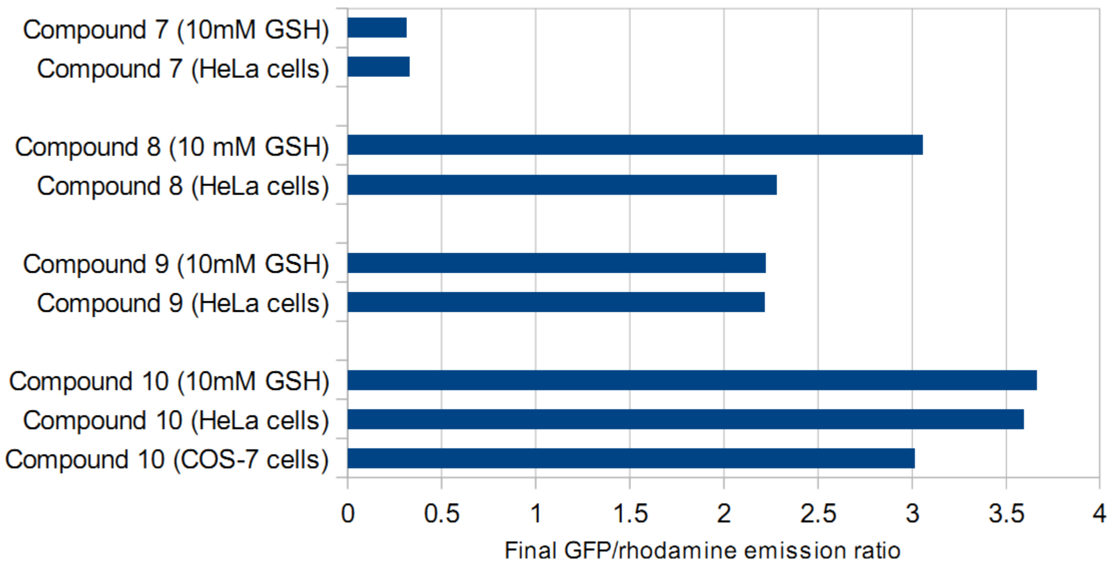

Figure S11. Ratio of final GFP/rhodamine emission intensities for cleaved compounds observed under the microscope. Microinjection values are an average from two injections. In vitro results are an average of three droplets.

11 References

1) Dubernet, M.; Caubert, V.; Guillard, J.; Viaud-Massuard, M.-C. *Tetrahedron* **2005**, *61*, 4585-4594.
2) Zimmer, M. *Chemical Reviews* **2002**, *102*, 759-782.
3) Rasband, W.S., ImageJ, U. S. National Institutes of Health, Bethesda, Maryland, USA, http://imagej.nih.gov/ij/, **1997-2011**.
